# “Expanding the Lactococcal Cell Wall Polysaccharide Paradigm: Novel Structures and Metabolic Pathways in the Emerging Dairy Species *Pseudolactococcus laudensis* and *Pseudolactococcus raffinolactis*”

**DOI:** 10.1002/mbo3.70133

**Published:** 2025-11-10

**Authors:** Axel Soto‐Serrano, Irina Sadovskaya, Evgeny Vinogradov, Wenwen Li, Jun‐Hyeok Yu, Kelsey White, Douwe van Sinderen, Lukasz Krych, Paulina Deptula, Jennifer Mahony

**Affiliations:** ^1^ Section for Food Microbiology, Gut Health and Fermentation, Department of Food Science University of Copenhagen Frederiksberg C Denmark; ^2^ Univ. Littoral Côte d'Opale, UMR 1158 BioEcoAgro, Institut Charles Viollette, USC ANSES, INRAe, Univ. Artois, Univ. Lille, Univ. Picardie Jules Verne, Univ. Liège Cedex 1 France; ^3^ National Research Council Canada Institute for Biological Sciences Ottawa ON Canada; ^4^ School of Microbiology & APC Microbiome Ireland University College Cork Cork Ireland

**Keywords:** bacteriophage (phage) receptor, cell wall polysaccharide (CWPS), exopolysaccharide (EPS), genomics, pseudolactococcus laudensis, pseudolactococcus raffinolactis

## Abstract

Cell surface‐associated polysaccharides, including cell wall polysaccharides (CWPSs), capsular polysaccharides (CPSs), and exopolysaccharides (EPSs), play vital roles in bacterial interactions with their environment, influencing critical aspects of dairy fermentations, such as phage–host dynamics. *Pseudolactococcus laudensis* and *Pseudolactococcus raffinolactis* (formerly *Lactococcus laudensis* and *Lactococcus raffinolactis*) are emerging dairy‐associated species whose CWPSs remain uncharacterized. This study analyzed the complete genomes of 21 *P. laudensis* and seven *P. raffinolactis* strains to investigate the genetic diversity underlying CWPS and EPS production. Eight novel *cwps* genotypes (E–L) were identified, significantly expanding the known diversity within the dairy‐associated (pseudo)lactococci. Notably, E and G genotypes diverge from the classical rhamnan‐PSP organization, suggesting a CWPS biosynthesis pathway distinct from the dual‐chain assembly found in previously studied *Lactococcus*. Additionally, *eps* loci were identified in 25 of the 28 strains, uncovering 11 distinct genotypes (I–XI) with evidence of horizontal gene transfer. Their integration into chromosomal genomic islands highlights their mobility and potential role in evolutionary adaptation. Chemical analysis revealed unprecedented CWPS structures. *P. laudensis* DSM 28961 (type E) presented a 6‐deoxy‐α‐l‐talan polysaccharide and a β‐(1,4)‐galactan, marking the first instance of d‐talose replacing rhamnose and the first homopolysaccharide in (pseudo)lactococcal CWPS, respectively. These were structurally independent, confirming a novel CWPS organization and biosynthetic pathway. Conversely, *P. raffinolactis* DSM 20443 (type I) exhibited a typical rhamnan‐PSP structure, composed of a variably glycosylated rhamnan and a glucose‐lactose hexapolysaccharide, respectively. This study provides the first resolved CWPS structures for the *Pseudolactococcus* genus, expanding the understanding of polysaccharide biosynthesis in Lactic Acid Bacteria.

## Graphical abstract

This study characterizes the genetic diversity of *Pseudolactococcus laudensis* and *Pseudolactococcus raffinolactis* regarding cell wall polysaccharides (CWPS) and exopolysaccharide (EPS) biosynthesis, uncovering novel genotypes and biosynthetic pathways. Structural analyses reveal unprecedented CWPS architectures, offering new insights into polysaccharide diversity and implications in dairy‐associated lactic acid bacteria.

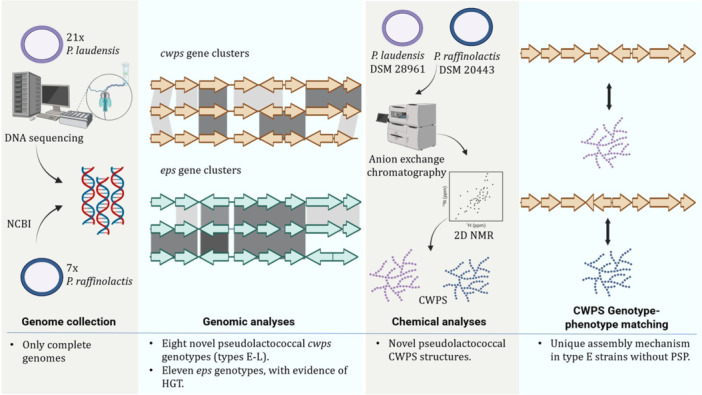

## 1 Introduction

Bacteria produce a range of intracellular and extracellular glycoconjugates, often exhibiting significant structural and functional diversity (Tytgat and Lebeer 2014). Among these, cell surface‐associated polysaccharides play critical roles in bacterial interactions with their environment. These polysaccharides are generally categorized into three types: exopolysaccharides (EPSs), which are loosely attached and released into the environment; capsular polysaccharides (CPSs), which are bound to the cell, forming a protective barrier; and cell wall polysaccharides (CWPSs), which may or may not be covalently linked to the cell wall but do not form a capsule (Chapot‐Chartier 2014). Among the industrially significant species *Lactococcus lactis* and *Lactococcus cremoris*, widely used in dairy fermentations, it is established that CWPSs serve as receptors for several lactococcal bacteriophage (phage) genera, including most commonly encountered Skunaviruses and the P335 group (Mahony et al. 2013, 2017). Given the economic impact of phage‐associated fermentation failure and delays (Guglielmotti et al. 2012), lactococcal CWPSs have been subject to extensive research. Consequently, the phage–host interactions of these two lactococcal species have, perhaps unsurprisingly, received the most significant research attention at the cost of other lactococcal species.

Recently, the genus *Lactococcus* was reclassified into two separate genera: *Lactococcus* and *Pseudolactococcus* (Abe et al. 2025). Currently, 26 species have been identified across these two genera, of which five are associated with milk or dairy environments: the well‐characterized *L. lactis* and *L. cremoris*, along with the emerging species *Lactococcus hircilactis, Pseudolactococcus laudensis*, and *Pseudolactococcus raffinolactis* (Abe et al. 2025; Mahony et al. 2023). In contrast to *L. lactis* and *L. cremoris*, little is known about these emerging species, despite their growing relevance in dairy microbiology (Mahony et al. 2023). Although some preliminary phenotypic characterizations exist (Jung et al. 2020; Klijn et al. 1995; Mahony et al. 2023; Meucci et al. 2015; Tidona et al. 2018), their CWPSs remain unexplored. Investigating these polysaccharides may uncover novel mechanisms of adaptation to dairy environments and provide insights into phage–host interactions, ultimately contributing to more robust fermentation systems. While the CWPSs of pseudolactococcal species have not been studied to date, the extensive analysis of those of *L. lactis* and *L. cremoris* has created an excellent foundation to characterize and compare their genetic and structural diversity.

Lactococcal CWPSs that have been characterized thus far consist of a neutral rhamnan component that is covalently attached to, and integrated within, the peptidoglycan layer, along with a surface‐exposed polysaccharide or polysaccharide pellicle (PSP) (Chapot‐Chartier et al. 2010; Mahony et al. 2013, 2020). Current existing models for CWPS biosynthesis include *L. cremoris* MG1363 (type C) (Theodorou et al. 2019) and *L. lactis* IL1403 (type B) (Mahony et al. 2020). In these, a dual biosynthetic pathway for both CWPS components is described, in which rhamnan is synthesized in the cytosol by the rhamnan precursor enzymes, RmlABCD and the rhamnosyltransferases RgpABFE, and transported to the outer part of the cell membrane via the ABC transporter system encoded by *rgpCD*. The PSP synthesis is also initiated in the cytosol by the priming glycosyltransferase, WpsA/YcbB, assisted by the membrane protein WpsB. The PSP is elongated and modified by a series of glycosyltransferases and modification enzymes, such as acetylases, epimerases, and mutases, among others. The PSP chain is subsequently translocated to the outer part of the cell membrane via the WpsG flippase and may or may not be polymerized depending on the presence or absence of the polymerase and co‐polymerase WpsI and WpsH (Theodorou et al. 2019). Finally, the PSP side‐chain is attached to the rhamnan moiety by the product of the gene *wpsJ*/*ycaFG* (a comprehensive figure illustrating this model is provided in Supporting Figure S1).

The CWPS components (PSP and rhamnan) are synthesized by a 20–30 kbp gene cluster known as the *cwps* cluster; however, some lactococcal strains possess glucose side chains attached to their rhamnan or PSP due to the activity of three‐component glycosylation systems (TGS) encoded in distinct loci from the *cwps* locus (Theodorou et al. 2020). The proximal (5’‐) region of the *cwps* cluster, which encodes rhamnan biosynthesis, is highly conserved, while the terminal (3’‐) region of the locus encodes PSP biosynthesis and displays significant genetic diversity. This variability likely contributes to the biochemical heterogeneity of CWPS structures, in which the PSP diversity underpins the narrow specificity of lactococcal phages (Mahony et al. 2020). Currently, all assessed lactococcal genomes harbor a *cwps* gene cluster that may be classified into one of four different genotypes (termed *cwps* A–D), with 11 subtypes within the C‐type clusters (Parlindungan et al. 2024).

CWPSs participate in several biological functions, including cell division (Theodorou et al. 2019) and cell wall biogenesis (Sadovskaya et al. 2017), defense against phagocytosis by macrophages (Chapot‐Chartier et al. 2010), and resistance against nonspecific host defense mechanisms (Reviewed by Guérin et al. 2022) in addition to their role in phage binding. Recent studies indicate that EPSs and CPSs also play a role in phage–host interactions involving lactococcal P335 phages (Millen et al. 2022), emphasizing the importance of a holistic approach in studying various cell surface‐associated polysaccharides.

EPSs are classified as homopolysaccharides (HoPS), composed of a single repeating monosaccharide, or heteropolysaccharides (HePS), which contain multiple monosaccharides and exhibit greater structural diversity due to variations in glycosidic bonds, molecular weight, and branching (Abarquero et al. 2022; Zhou et al. 2019). The synthesis of HoPS typically occurs via the dextrase/sucrase‐dependent pathways, whereas heteropolysaccharidic EPS and CPS in Gram‐positive bacteria are mainly synthesized *en bloc* via the Wzx/Wzy pathway (Tytgat and Lebeer 2014). The genes governing HePS biosynthesis are co‐located in the so‐called *eps* gene clusters, which typically range from 11 to 22 kbp and are variably present in the chromosome or in plasmids in different species (Zeidan et al. 2017). The *eps* clusters harbor genes associated with polysaccharide assembly (initiation *epsE*, polymerization *wzy*, export/flippase *wzx*, and attachment *epsA*), modulatory genes (phosphoregulatory module *epsBCD*), glycosyltransferase‐encoding genes, and genes required for synthesizing activated sugar precursors and the modification of the sugar residues (Cui et al. 2023; Zhou et al. 2019). The genes *epsABCDE* are located in the proximal region and are highly conserved, while the remainder of the cluster exhibits significant genetic variability. Additionally, *L. lactis* contains the conserved gene of unknown function *epsX* in the proximal region, and the gene of unknown function *epsL* and the *orfY* modulator represent a second conserved region at the terminal end (Zeidan et al. 2017). Less common pathways for the synthesis of EPS and CPS include the synthase‐dependent pathway, as well as ABC transporter‐dependent pathways commonly reported for Gram‐negative bacteria (Schmid 2018); however, none of these have so far been described in *Lactococcus*.

EPSs and CPSs may provide protection against environmental stresses, such as low pH or antimicrobial compounds (Caggianiello et al. 2016). Furthermore, they may promote biofilm formation and support probiotic activity by enhancing adhesion, pathogen resistance, and immune modulation (Zhou et al. 2019). Finally, EPSs are highly valued in the food industry due to their water‐retention and stabilizing effects, with the ability to improve the texture, viscosity, and stability of food products (Abarquero et al. 2022; Zhou et al. 2019).

The aim of this study was to investigate and characterize the genetic and structural diversity of cell surface‐associated polysaccharides in the recently reclassified dairy‐associated species *P. laudensis* and *P. raffinolactis*. To this end, the *cwps* and *eps* clusters of 21 *P. laudensis* and seven *P. raffinolactis* strains were identified and compared within and across pseudolactococcal, lactococcal, and other lactic acid bacteria species. Additionally, the CWPS structures of the reference strains *P. laudensis* DSM28961 and *P. raffinolactis* DSM 20443 (KACC13441) were resolved using nuclear magnetic resonance (NMR) spectroscopy.

This study represents the first cell wall‐associated polysaccharide analysis of the genus *Pseudolactococcus*, expanding current knowledge of the diversity and commonalities between the closely related genera *Lactococcus* and *Pseudolactococcus,* and providing foundational insights into polysaccharide biosynthesis pathways with potential implications for dairy fermentations and phage–host interactions.

## 2 Materials and Methods

### 2.1 DNA Extraction and Sequencing

*P. laudensis* isolates were obtained from a commercial source (DSM 28961, DSMZ, Braunschweig, Germany) or an undefined mesophilic starter culture (Li et al. 2024) after plating on M17 agar (Oxoid, Hampshire, UK) supplemented with 0.5% lactose (Merck, Darmstadt, Germany) (LM17 0.5%) at 30°C for 48 h. Single colonies were transferred into LM17 1% broth and incubated at 30°C for 48 h. The cultures were centrifuged at 4,402 × *g* for 10 min in a 5920 R centrifuge (Eppendorf, Hamburg, Germany). The supernatants were discarded, and pellets were resuspended in 1 mL of 0.9% NaCl, transferred into 1.5 mL tubes (Eppendorf, Hamburg, Germany), and centrifuged using a Micro Star 17 R microfuge (VWR, Radnor, USA) at 12,000 × *g* for 3 min. Genomic DNA was extracted from the resulting pellet utilizing the Bead‐Beat Micro AX 283 Gravity kit (cat # 106‐100‐M1, A&A Biotechnology, 284 Gdynia, Poland) following the instructions of the manufacturer. DNA concentration was measured utilizing a Qubit 4 fluorometer (Thermofisher, Waltham, USA) and normalized to 33.3 ng/μl (400 ng total) for library preparation using the Native Barcoding Sequencing protocol (SQK‐NBD114.24, Oxford Nanopore Technologies, Oxford, UK) following the manufacturer's instructions. Sequencing was performed on a PromethION 2 Solo platform. Basecalling was conducted with Dorado v0.7.1 (https://github.com/nanoporetech/dorado) using the dna_r10.4.1_e8.2_400bps_sup@v5.0.0 basecalling model.

### 2.2 Genome Assembly and Reorientation

Raw data were filtered using NanoFilt v2.6.0 (De Coster et al. 2018) to a minimum quality of 8 and a minimum length of 1,000 bp. Assembly was performed utilizing Trycycler v0.5.4 (Wick et al. 2021) with the data obtained after splitting the reads into 12 subsets and assembling those with Flye v2.9.1 (Kolmogorov et al. 2019), Canu v2.2 (Koren et al. 2017), and Hybracter v0.9.0 (Bouras et al. 2024b) for optimized plasmid recovery (Bouras et al. 2024b; Johnson et al. 2023).

The complete genomes from the *P. raffinolactis* strains were obtained from the National Center for Biotechnology Information (NCBI).

All chromosome and plasmid contigs in this study, including those from *P. raffinolactis* obtained from NCBI, were reoriented with dnaapler v0.8.1 (Bouras et al. 2024a) to start with *dnaA* or *repA*, respectively.

The accession numbers of the genomes utilized in this study are indicated in Supporting Table S1.

### 2.3 Identification and Characterization of the *cwps* and *eps* Clusters

For the identification of the *cwps* and *eps* clusters, the genomes of the *P. laudensis* and *P. raffinolactis* strains were annotated utilizing Bakta v1.9.4 (Schwengers et al. 2021) and subjected to nucleotide Basic Local Alignment Search Tool (BLAST) (Camacho et al. 2009) analysis against well‐characterized reference clusters. Specifically, *cwps* clusters from *L. lactis* IL1403 (accession number CP033607.1, positions 196221 to 220264 bp) and *L. cremoris* MG1363 (accession number AM406671.1, positions 194607 to 218654 bp) were selected as they represent model strains with extensively studied *cwps* loci and are representatives of two of the three major *cwps* genotypes established to date, that is, *cwps* B and C, respectively (Mahony et al. 2020; Theodorou et al. 2019). Likewise, the *eps* cluster from *L. lactis* KLDS 4.0325 (accession number CP006766.1, positions 84302 to 101534 bp) was selected due to its complete annotation (Zeidan et al. 2017). Annotation files were manually curated for downstream analyses.

Nucleotide and amino acid sequence similarities were calculated using BLAST (Camacho et al. 2009), and these are reported in the manuscript as the product of Query Cover × Percentage Identity values. Gene cluster similarities and alignments were visualized using Clinker (Gilchrist and Chooi 2021) via the CAGECAT webserver, release 1.0 (van den Belt et al. 2023). Additionally, protein structure prediction with DeepTMHMM v1.0 (Hallgren et al. 2022), as well as analyses with HHPred v 57c8707149031cc9f8edceba362c71a3762bdbf8 (Hildebrand et al. 2009), Conserved Domain Database (CDD) v3.21 (Wang et al. 2023), and Prokka v1.14.6 (Seemann 2014) were performed to further characterize the *cwps* and *eps* genes/gene products.

To characterize the genetic context of both *cwps* and *eps* loci, the genomes of the *P. laudensis* and *P. raffinolactis* strains were analyzed with Phastest v3.0 (Wishart et al. 2023) to determine the presence of prophages and IslandCompare v1.0 (Bertelli et al. 2022) for the identification of other potential genomic islands. Furthermore, regions flanking the *cwps* and *eps* loci were analyzed with ICEfinder from the ICEberg 3.0 webserver (Wang et al. 2024) and mobileOG‐db v1.1.3 (Brown et al. 2022) through the Proksee webserver (Grant et al. 2023).

### 2.4 Extraction and Purification of Polysaccharides and Their Fragments

#### 2.4.1 *Pseudolactococcus laudensis* DSM 28961

Cells derived from ~1.3 L of a fresh overnight culture of *P. laudensis* strain DSM 28961, grown in LM17 1% media, were harvested by centrifugation at 5,000 × *g* for 15 min and treated by autoclaving (120°C, 30 min). The cell debris was harvested by centrifugation (yielding ~11 g of debris) and treated first in 5% trichloroacetic acid (TCA) in water at 5°C for 48 h. Subsequently, the sample was treated with hot 0.01 M hydrochloric acid (HCl) for 20 min under stirring in a boiling water bath (100°C), followed by cooling, centrifugation at 12,000 x *g,* and resuspendion in 0.1 N HCl for a second 20 min treatment under the same conditions (Sadovskaya et al. 2017; Parlindungan et al. 2024). This approach facilitates the extraction of fractions enriched with either “outer cell wall (CW) layer” PSP (cold TCA extract) or “inner CW layer” rhamnan (often 0.1 M HCl extract). The following yields of crude extracts were obtained: autoclaved (AU) extract—200 mg; TCA extract—100 mg; 0.01 M HCl extract ~100 mg; 0.1 M HCl extract—46 mg. All extracts contained l‐dTal (6‐deoxytalose), d‐Glc (glucose), and d‐Gal (galactose) in different ratios as major monosaccharide components. The AU extract also contained arabinose. Traces of arabinose and xylose were present in other extracts. AU and 0.1 M HCl extracts were further fractionated by anion‐exchange chromatography. After desalting, fractions were screened by monosaccharide analysis and ^1^H‐NMR spectroscopy. Two CWPS fractions named CWPS1 and CWPS2 were eluted with ~ 0.1 M NaCl and 0.2–0.3 M NaCl, respectively. The neutral fraction contained a mixture of CWPS1 and CWPS2. A portion of CWPS1 was treated with NH_4_OH (50°C, 30 min) and desalted on a Sephadex G‐15 column to yield the de‐*O*‐acetylated CWPS1 (DPS).

#### 2.4.2 *Pseudolactococcus raffinolactis* DSM 20443

The cell pellet derived from 4 L of a fresh overnight culture of *P. raffinolactis* DSM 20443 grown in tryptic soy yeast extract (TSB) broth supplemented with 0.3% yeast extract was first extracted by autoclaving (120°C, 30 min). Cell debris was removed by centrifugation, and the supernatant was deproteinated through the addition of 5% TCA, dialyzed, and lyophilized, yielding the crude AU extract (110 mg). Cell debris (~5 g) was subjected to consecutive acid extractions as described previously (Parlindungan et al. 2024), yielding a crude TCA extract (3 mg), a 0.01 M HCl extract (50 mg), and a 0.1 M HCl extract (45 mg).

Upon fractionation on a Sephadex G‐50 column, HCl extracts yielded one homogeneous peak (rhamnan) containing l‐Rha (rhamnose) and d‐Glc in an approximate ratio of 2.3:1 and trace amounts of GlcNAc (N‐acetyl glucosamine) and d‐Gal.

The AU extract eluted into two fractions: a high molecular weight (HMW) fraction with a composition identical to the rhamnan from the HCl extract and a low molecular weight (LMW) fraction, containing d‐Glc and d‐Gal in an approximate ratio of 1.3:1. The LMW fraction was further purified by anion‐exchange chromatography on Q‐Sepharose. The major fraction, eluted at the beginning of the gradient, was collected and desalted on a Biogel P2 column and named OS1 (Oligosaccharide 1).

### 2.5 General and Analytical Methods

Chromatography, monosaccharide and methylation analysis, electrospray ionization mass spectrometry (ESI‐MS), and NMR spectroscopy were performed as described previously (Mahony et al. 2020; Parlindungan et al. 2024). The absolute l‐configuration of 6‐deoxy‐d‐talose (6dTal) was determined by GC‐MS of the acetylated (R)‐(‐)‐2‐butyl glycoside derivative by comparison with an authentic standard. The O‐acetylated homopolysaccharide (OPS) from *Pseudomonas* (*Burkholderia*) *plantarii* DSM 6535 was used as the source of D‐6dTal (Zähringer et al. 1997).

## 3 Results

### 3.1 Identification of *cwps* Clusters in *P. laudensis* and *P. raffinolactis* Genomes

The genomes of 21 *P. laudensis* strains were sequenced and assembled in the context of this study to investigate genes involved in cell surface‐associated polysaccharide biosynthesis. All assemblies yielded complete genomes comprising a circular chromosome and a variable number of circular plasmids. Additionally, the analysis included all seven *P. raffinolactis* genomes currently available as complete assemblies in public databases (NCBI).

**Figure 1:**
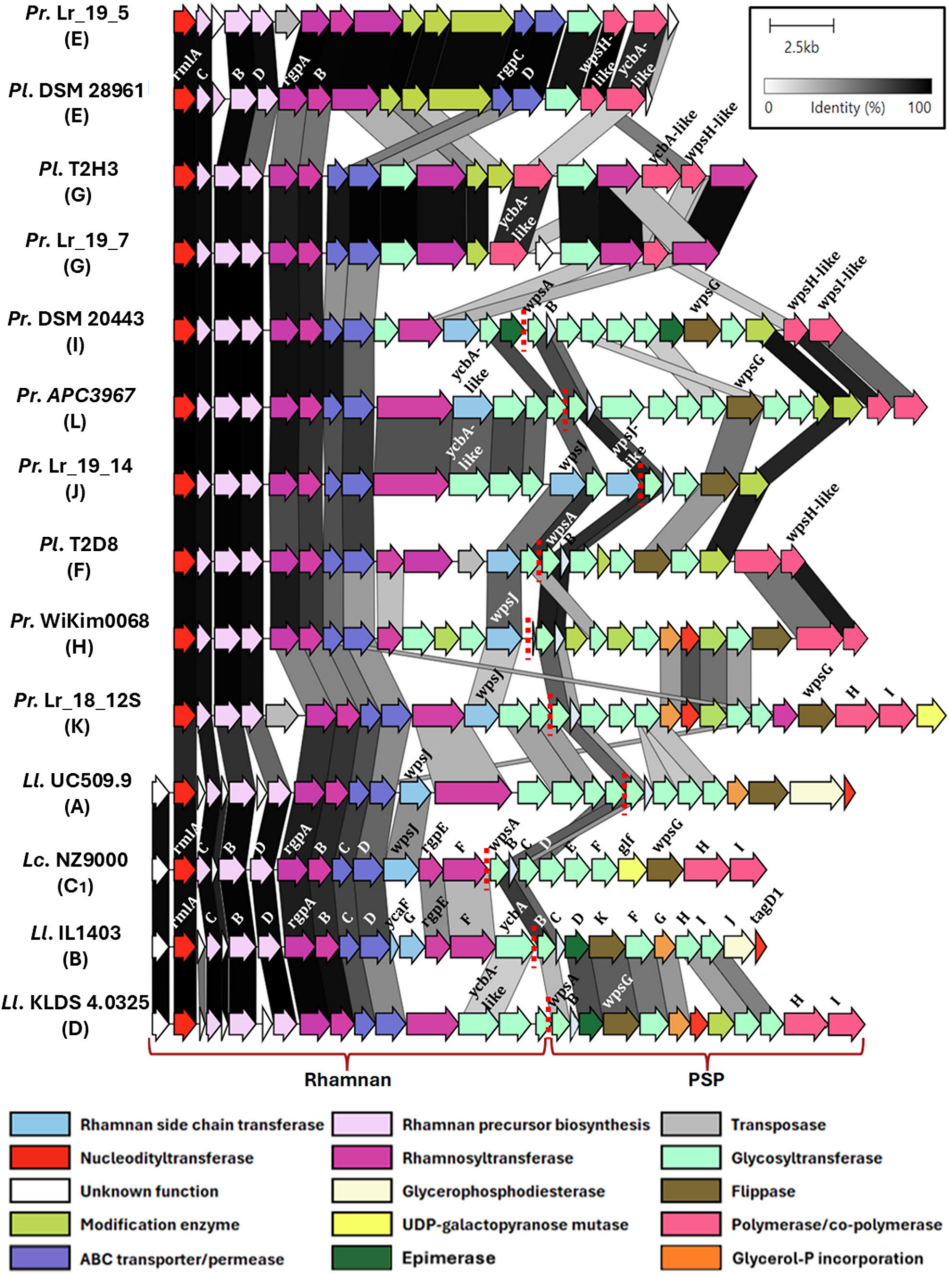
Overview of the organization and sequence similarity of the *cwps* gene clusters of *P. laudensis (Pl.), P. raffinolactis (Pr.)* types E‐L, and selected *L. lactis (Ll.) and L. cremoris (Lc.)* types A, B, C, and D. The proposed functions for each of the genes are color‐coded and have been inferred by gene annotation, as well as sequence and structure similarity. Red dashed lines separate the rhamnan (left) and PSP (right) encoding regions of the clusters.

The *cwps* clusters from all *P. laudensis* and *P. raffinolactis* strains were identified and extracted for further genomic analysis. All retrieved genomes were found to harbor a *cwps* cluster ranging in size from 18,445 to 30,217 bp and encoding between 16 and 28 genes. The strains were subsequently classified into genotypes based on their similarity regarding the PSP‐encoding region of their *cwps* cluster, that is, the terminal region of the cluster, starting from *wpsA*, which is predicted to encode the priming glycosyltransferase. The following criteria were used to assign the strains into different *cwps* genotypes: (i) the presence or absence of polymerase‐ and co‐polymerase‐encoding genes, (ii) the presence or absence of a flippase‐encoding gene, and (iii) the occurrence of two or more PSP‐encoding genes—excluding the flippase, polymerase, and co‐polymerase—sharing < 30% amino acid similarity with another genotype. Based on these criteria, eight novel *cwps* genotypes were identified: five exclusive to *P. raffinolactis*, one unique to *P. laudensis*, and two shared by a single strain of each species. A visual representation comparing the (pseudo)lactococcal *cwps* clusters is provided in Figure 1, highlighting the sequence similarity and unique regions of the respective clusters. The comparative analysis allowed the identification of novel genotypes using similar approaches to previous studies of lactococcal *cwps* genotyping, that is, the presence of at least one distinct glycosyltransferase‐encoding gene, which has been established to be associated with distinct saccharidic composition/structures. This analysis revealed a broader diversity of CWPSs in *P. laudensis/raffinolactis* than in *L. lactis/cremoris*, in which only four genotypes are described (A‐D) with similar classification criteria (Mahony et al. 2020), despite a greater diversity of the latter regarding their isolation sources. In that sense, all the *P. laudensis* strains except the reference DSM 28961 were isolated from the same commercial starter culture (Li et al. 2024; this study).

Among the studied strains, *P. raffinolactis* Lr_18_12S was shown to exhibit the greatest similarity to known *L. lactis* and *L. cremoris cwps* genotypes, particularly to type A *L. lactis* UC509.9 (Figure 1). These findings, along with shared genotypes between *P. laudensis* and *P. raffinolactis*, suggest that additional shared genotypes may exist in other *Lactococcus/Pseudolactococcus* species. Thus, the novel genotypes were designated E–L, continuing the established *cwps* nomenclature. Table 1 details the distribution of genotypes, intra/inter‐species prevalence, as well as the genomic locations and length of the *cwps* clusters.

**Table 1:** Genomic location, genotype, and size of *cwps* and *eps* clusters for the *P. laudensis* and *P. raffinolactis* strains.

		CWPS				EPS			
Species	Strain	*cwps* genotype	Cluster start (bp)	Cluster end (bp)	Cluster length (bp)	*eps* genotype	Cluster start (bp)	Cluster end (bp)	Cluster length (bp)
*P. laudensis*	DSM28961	E	182887	201332	18445	V	74320	90617	16297
	T2A4	F	1999196	2023901	24705	I	79289	93569	14280
	T2A6	F	1946019	1970724	24705	VI	75286	94233	18947
	T2A7	F	2085468	2110175	24707	I	107107	121389	14282
	T2A8	F	2017410	2042116	24706	I	79288	93568	14280
	T2C1	F	2022619	2047325	24706	IV	74431	91778	17347
	T2C6	F	1944846	1969552	24706	VI	75286	94233	18947
	T2C9	F	2042130	2066835	24705	I	79288	93568	14280
	T2D8	F	2054139	2078844	24705	I	79288	93568	14280
	T2E11	F	150314	175019	24705	—	—	—	—
	T2E12	F	1944854	1969559	24705	VI	75286	94233	18947
	T2E8	F	2054342	2079047	24705	I	79289	93569	14280
	T2F10	F	1944795	1969499	24704	VI	75286	94233	18947
	T2F2	F	1944847	1969552	24705	VI	75286	94233	18947
	T2F8	F	2052966	2077672	24706	I	79288	93568	14280
	T2G11	F	2048104	2072809	24705	III	74437	90237	15800
	T2G3	F	2020537	2045242	24705	IV	74430	91777	17347
	T2G5	F	2104178	2117025	12847	VII	76449	90201	13752
			295724	307558	11834				
	T2H1	F	1999389	2024094	24705	VI	75287	94234	18947
	T2H3	G	2031233	2054047	22814	II	78886	98046	19160
	T2H4	F	2104194	2117041	12847	VII	76449	90202	13753
			295725	307559	11834				
*P. raffinolactis*	WiKim0068	H	183549	210693	27144	VIII	80195	94639	14444
	DSM 20443	I	167655	193821	26166	IX	61407	78976	17569
	Lr_19_7	G	231981	253318	21337	X	102387	125579	23192
	Lr_19_5	E	374429	394173	19744	—	—	—	—
	Lr_19_14	J	173936	197235	23299	XI	1151222	1173701	22479
	Lr_18_12S	K	168009	198226	30217	XI	1851815	1874084	22269
	APC3967	L	152269	181745	29476	—	—	—	—

The most prevalent *cwps* genotype among the assessed strain collection was type F, present in 19 of the 21 *P. laudensis* strains. While these strains exhibit practically identical nucleotide composition in their *cwps* clusters, deeper analysis revealed transposase insertions at varying sites, disrupting the predicted *wpsJ* gene—responsible for transferring the PSP side chain onto the rhamnan backbone—and a transmembrane modification enzyme (Fig. S2). Additionally, the *cwps* cluster in strains T2H4 and T2G5 was found to be split into two in distant sites within the chromosome, most certainly due to the action of the aforementioned transposase, since it moved the genes from its position to the next transposase situated downstream of the *cwps* cluster. The functional consequences of these insertions and rearrangements on CWPS biosynthesis remain to be experimentally validated; however, their consistent occurrence in strains from the same starter culture may reflect selective pressure to modify or eliminate the PSP component, likely as an adaptive response to phage predation or niche adaptation to the processing environment. Regarding the other *cwps* genotypes, E and G were each present in a strain of both *P. laudensis* and *P. raffinolactis*, while the rest were unique to single strains (Table 1).

Similar to the previously studied *L. cremoris* and *L. lactis*, the proximal region of the clusters containing the deoxy‐thymidine‐disphospho‐rhamnose (dTDP‐Rha) precursor genes *rmlABCD*, the rhamnosyltransferases *rgpAB*, and the ABC transporter system *rgpCD* is highly conserved among all the lactococcal strains, though the latter genes are located further in the cluster in type E strains. Conversely, the PSP‐encoding regions exhibited high variability among the different clusters, especially in the number and composition of glycosyltransferases, other transferases, and modification enzymes (Figure 1).

### 3.2 Functional Predictions of the PSP Side Chain‐Encoding Regions of the *cwps* loci

The PSP priming glycosyltransferase WpsA, its assisting membrane anchor WpsB, the flippase WpsG, responsible for PSP translocation across the cell membrane, and WpsJ, involved in attaching the PSP to the rhamnan backbone (Theodorou et al. 2019), play essential roles in the PSP biosynthesis. In most *Pseudolactococcus* strains analyzed, these genes were either conserved or could be confidently inferred based on TMHMM modeling predictions or HHPred structural homology predictions (Theodorou et al. 2019) (Figure 1; Supporting Data). Specifically, when nucleotide or amino acid similarity was insufficient for identification, *wpsJ* and *wpsG* were inferred based on analysis of gene products with transmembrane DUF2142‐containing proteins and predicted structures with 12–14 transmembrane helices (TMHs), respectively (Hvorup et al. 2003; Kuk et al. 2022). However, in genotypes E and G, neither sequence homology nor structural predictions enabled identification of *wpsA*, *wpsB*, *wpsG*, or *wpsJ* homologs, strongly suggesting that these genotypes utilize an alternative CWPS assembly mechanism, distinct from the dual biosynthetic pathway previously characterized in *Lactococcus*.

Among the studied strains, only *P. raffinolactis* Lr_18_12S (type K) harbored gene products with sequence similarity to the putative WpsI and WpsH oligosaccharide polymerase and co‐polymerase proteins from *L. lactis and L. cremoris* type C and D strains. DeepTMHMM analysis confirmed these findings, predicting protein structures identical to those of *L. cremoris* NZ9000 (type C_1_): WpsI containing a DUF2142 domain and 11 TMHs, while WpsH featuring a large loop in the outer part of the cell membrane flanked by a signal peptide and a TMH in the N‐ and C‐termini, respectively.

DeepTMHMM analysis also revealed WpsI‐ and WpsH‐like candidates in several other strains. Specifically, a gene annotated by Bakta as a bactoprenol glycosyltransferase exhibited two TMHs at the terminal end, resembling the WpsH co‐polymerase structure, which contains a single TMH instead. This gene was found at the 3’‐end of the corresponding *cwps* loci, adjacent to genes annotated as glycosyltransferases containing a DUF2142 and 10‐12 TMHs, which we hypothesize encode the polymerase function. The presence of these genes in all *P. laudensis* and *P. raffinolactis cwps* genotypes, except in type J, suggests that the strains produce polymeric PSP, as is the case for the type C and D *L. lactis* and *L. cremoris* strains studied to date (Mahony et al. 2020; Parlindungan et al. 2024; Theodorou et al. 2019). In contrast, in type J, the PSP is likely an oligomer.

Among the identified DUF2142 domain‐containing putative polymerases, three groups can be distinguished: (i) proteins with lactococcal genotype C and D WpsI‐like predicted structures, although with low amino acid sequence similarity, found in types I and L; (ii) proteins containing an ALG6/ALG8 domain, identified as by CDD in type F strains; and (iii) proteins containing a Protein O‐Mannosyltransferase 2 superfamily domain as indicated by HHPred and CDD analyses, present in the type E and G clusters. The latter of these three putative polymerase groups exhibits significant structure similarity with YcaA from the type B *L. lactis* IL1403, the only gene product within this cluster that has not yet been functionally assigned (Mahony et al. 2020). This raises the possibility that YcaA encodes a polymerase with limited or defective activity due to the absence of an associated co‐polymerase. Other YcaA‐like proteins are present in several of the *cwps* loci, in variable locations within the clusters (Figure 1). Alternatively, YcaA‐like proteins could be involved in glycan modification, since Protein‐O‐mannosyltransferases are conserved among domains of life and known to be responsible for protein O‐mannosylation (Lommel and Strahl 2009). Finally, the structure of WpsJ is very similar to this of the WpsI polymerase in type C and D strains, such as *L. cremoris* NZ9000. Thus, it is possible that these DUF2142‐containing YcaA‐like proteins function as WpsJ PSP‐tranferases in strains including *L. lactis* KLDS4.0325 and *P. raffinolactis* APC3967, given the lack of other candidates performing such functions (Figure 1). Experimental validation is required to confirm the putative functions of these genes, which is beyond the scope of the present study.

### 3.3 Identification of the *eps* Clusters

**Figure 2:**
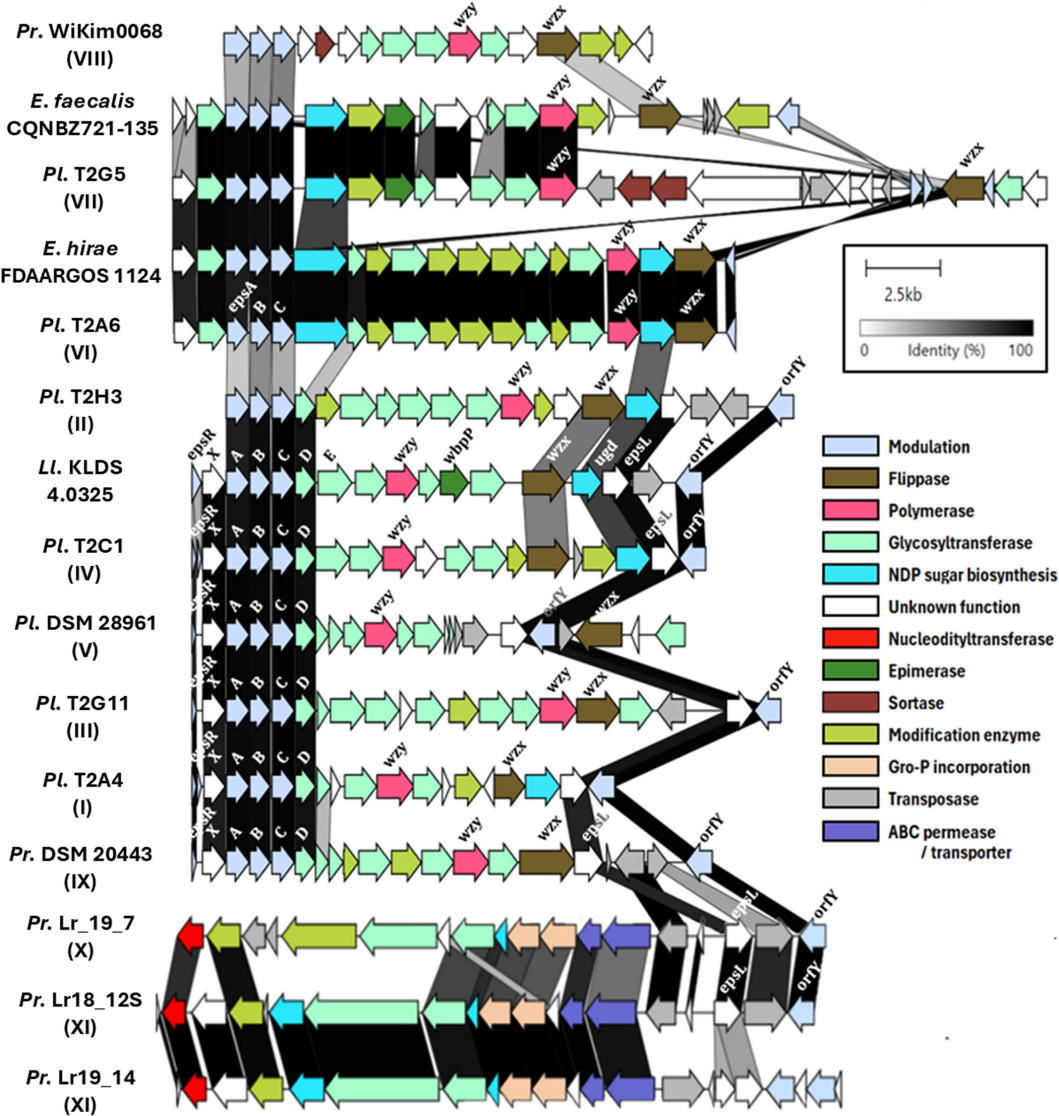
Overview of the organization and sequence similarity of the *eps* gene clusters of *P. laudensis (P.) and P. raffinolactis (Pr.)* types I‐XI, *L. lactis* (Ll.) KLDS 4.0325, and selected *Enterococcus faecalis* and *Enterococcus hirae* strains. The proposed functions for each of the genes are color‐coded and have been inferred by gene annotation, as well as sequence and structure similarity.

The recent discovery of EPS‐dependent lactococcal phage–host interactions (Millen et al. 2022) accentuates the need to adopt a holistic approach when studying cell surface‐associated polysaccharides among lactococci and beyond. Among the studied strains, all except *P. laudensis* T2E11 and *P. raffinolactis* Lr_19_5 and APC3967 were found to harbor an *eps* cluster. A schematic representation of the *eps* clusters is presented in Figure 2. Based on sequence similarity, 11 distinct genotypes (I–XI) were identified. The distribution of these genotypes, along with the length and genomic location of the *eps* clusters, is detailed in Table 1.

The *epsRXABCD* genes at the proximal end of the cluster are conserved among the I, III, IV, V, and IX genotypes, whereas genotypes II, VI, VII, and VIII contain only phosphoregulatory *epsABC* genes (Figure 2). Within these genotypes, types I, II, and IV possess a flippase‐encoding gene with similarity to the *wzx* in *L. lactis* KLDS 4.0325, while all other types possess a gene encoding a protein with the typical membrane topology and characteristics of flippases (i.e., 12‐14 transmembrane helices) from the multidrug/oligosaccharidyl‐lipid/polysaccharide (MOP) exporter superfamily (Hvorup et al. 2003; Kuk et al. 2022). Bakta annotations revealed the presence of genes encoding the likely *wzy* polymerase within the gene clusters of *eps* genotypes V and VII, and DeepTMHMM enabled the inference of genes encoding transmembrane polymerases for genotypes I‐IX, confirming those of the V and VII types (Figure 2; Supporting Data). The presence of the regulatory‐associated genes, in addition to the flippase‐ and polymerase‐encoding genes, indicates that the strains belonging to the I‐IX genotypes likely synthesize heterosaccharidic EPS *en bloc* via the Wzy‐dependent pathway (Tytgat and Lebeer 2014).

In contrast, genotypes X and XI harbor an ABC transporter system, lack the signature genes of the Wzx/Wzy pathway, and share gene similarity with genotypes I–IX only in the *epsL* gene of unknown function and the *orfY* modulator (Figure 2). The Wzy‐independent or ABC transporter‐dependent pathway is associated with the sequential synthesis and export of HePS, which remain bound to the cell envelope, that is, CPS, in Gram‐negative bacteria (Schmid 2018; Whitfield et al. 2024). To the best of our knowledge, no ABC transporter‐dependent pathways have been reported for the synthesis of EPS or CPS in Gram‐positive bacteria.

The different *eps* genotypes exhibit an elevated degree of variability regarding their glycosyltransferases and modification enzymes composition. For example, strains like T2G11 (type III) encode 8 different glycosyltransferases and only a single modification enzyme annotated as a putative phosphotransferase. Conversely, type VI strains possess 4 glycosyltransferases and diverse modification enzymes, including putative acetyltransferases, dehydratases, and epimerases (Figure 2). Such genetic variability among the different genotypes is presumed to underlie the potential structural diversity among the synthesized EPS and/or CPS; however, it remains to be proven that this genetic potential is linked to the actual production of EPS under laboratory or food production conditions.

Interestingly, *eps* genotypes VI and VII exhibit an elevated sequence similarity to *eps* clusters from certain *Enterococcus* strains (Figure 2). Nucleotide BLAST revealed > 99.5% nucleotide similarity between type VI clusters and *Enterococcus hirae* FDAARGOS_1124 and *E. hirae* FDAARGOS_1123 across the entire cluster, as well as > 97% nucleotide similarity between type VII and *Enterococcus faecalis* CQNBZ21‐135 and *E. hirae* FDAARGOS_1057, spanning from the start of the cluster to the Wzy‐like polymerase‐encoding gene (Figure 2). These *E. faecalis* and *E. hirae* strains were isolated in feces in 2021 in China and from an unknown source in Germany, respectively. Given their sequence similarity, and the variability of glycosyltransferases and modification enzymes encoded by *eps* clusters, it is unlikely that these clusters evolved independently in strains from different genera.

The intriguing similarity between the *eps* loci of *P. laudensis* and the aforementioned *Enterococcus* strains motivated further investigation regarding the genetic context of these clusters, with the aim of elucidating their evolutionary origins and potential horizontal gene transfer (HGT) events. Similarly, exploring the genetic context of the *cwps* clusters may reveal additional insights into their functional organization and mobility.

### 3.4 Genetic Context of the *eps* Gene Clusters

Both *eps* and cw*ps* gene clusters of all analyzed *P. laudensis* and *P. raffinolactis* strains were observed to be chromosomally located. The finding of chromosomally encoded *eps* loci in these species is in contrast to the majority of *L. lactis/cremoris eps* loci, which are more commonly plasmid‐associated (Parlindungan et al. 2024). To characterize their genetic context and explore their possible origin and mobility, the genomic regions flanking both loci were analyzed.

Bakta and mobileOG‐db annotations suggested a HGT origin for the *eps* clusters. In all *P. laudensis* and *P. raffinolactis* strains, the regions directly up‐ and downstream of the *eps* cluster contained several genes annotated as phage and plasmid‐associated genes, transposases, and conjugal transfer proteins. Additionally, these regions also contained putative antimicrobial resistance genes (i.e., Enterocin A immunity) and phage defense systems (e.g., AbiV in T2C1 or T2A4), commonly associated with HGT (Makarova et al. 2011).

**Figure 3:**
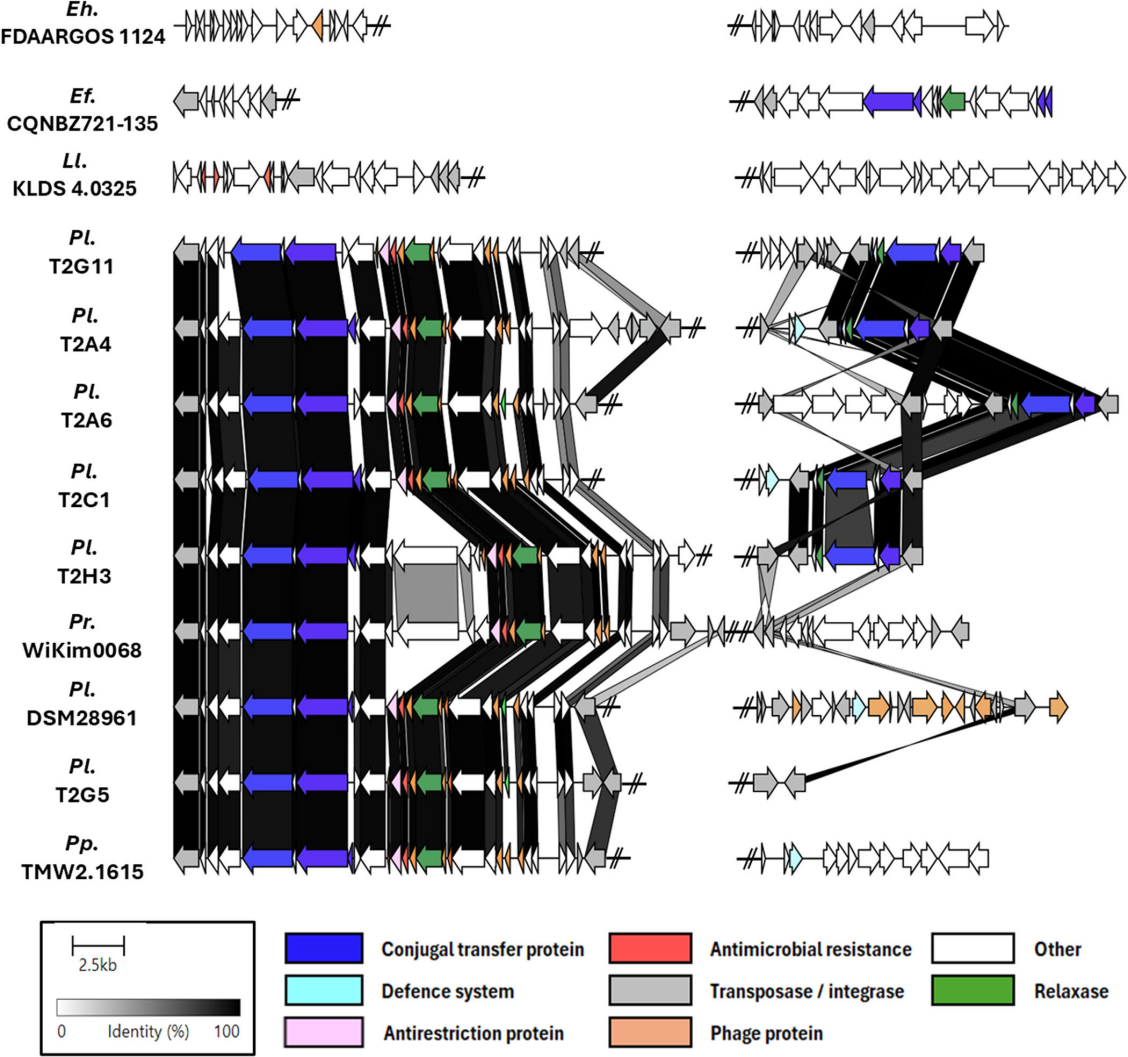
Genetic context of the *eps* cluster in selected lactococcal and enterococcal strains, showing (left) the upstream region and (right) the downstream region. For the *P. laudensis* (*Plau*.), *P. raffinolactis* (*Lr*.), and *P. paracarnosus* (*Pp*.) strains, the regions represent genomic islands. For *L. lactis* (*Ll*.) KLDS 4.0325 and *E. hirae* (*Eh*.) and *E. faecalis* (*Ef*.) strains, the size of the flanking regions was decided arbitrarily. The *eps* cluster has been manually removed for better visualization of the up‐ and downstream regions.

IslandCompare analysis confirmed that the *eps* loci, along with adjacent regions, constitute genomic islands integrated into the lactococcal chromosomes. After manual curation, genomic islands were determined to range in size from ~45 to 55 kbp, with the upstream regions consistently larger than the downstream regions (Figure 3). For all the *P. laudensis* strains, as well as *P. raffinolactis* WiKim0068 and DSM 20443, these genomic islands were integrated in the same location within their chromosome, as can be observed in Table 1 by the location of the *eps* cluster. Interestingly, the island was conserved in all *P. laudensis* strains, including T2E11, which lacks an *eps* cluster.

Nucleotide BLAST analysis revealed elevated sequence similarity between the genomic islands of all *P. laudensis* and *P. raffinolactis* strains, particularly in the upstream regions (80–98% identity). Interestingly, a comparable level of similarity was observed with a chromosomal region in the meat isolate *Pseudolactococcus paracarnosus* TMW 2.1615, the only strain of its species with a complete genome available in NCBI (Figure 3). Notably, this region also precedes an *eps* gene cluster in *P. paracarnosus*.

Further analysis with Phastest indicated that the *eps* upstream flanking regions of *P. laudensis* and *P. raffinolactis* strains contain a putative incomplete prophage, yet the predictions were inconsistent between the strains despite their sequence similarity. Moreover, in *P. laudensis* DSM 28961, the *eps* downstream region was identified as an intact prophage. However, the latter does not present a typical prophage structure (i.e., no apparent lysis, structural, or replication modules), and most annotated phage genes comprise transposases, along with a couple of glycosyltransferases, suggesting a false positive result from Phastest from what appears to be a mobile genetic element (MGE). Notably, the insertion site of the MGE is within the *eps* cluster, specifically downstream of the second transposase (Figure 2). This insertion modifies the genetic composition of the *eps* cluster and likely influences EPS composition in this strain. A comparable mechanism occurs in pathogenic bacteria such as *Shigella flexneri*, where prophage‐encoded glycosylation or acetylation modifies the O‐antigen, a process known as serotype conversion (Jakhetia et al. 2013; Markine‐Goriaynoff et al. 2004).

Finally, ICEfinder analysis identified the upstream and downstream regions of the *eps* clusters as integrative conjugative elements (ICEs) and integrative mobile elements (IMEs), respectively. Although the upstream regions contain genes encoding a predicted relaxase, a type IV secretion system (T4SS), and a type IV coupling protein (T4CP)‐encoding gene, they appear to lack an origin of transfer (OriT), preventing their self‐transmission.

The *eps* clusters of *E. hirae* FDAARGOS_1124, FDAARGOS_1123, and FDAARGOS_1057 and *E. faecalis* CQNBZ21‐135 were analyzed to investigate their genetic context. In *E. hirae* FDAARGOS_1124 and FDAARGOS_1123, the identified *eps* clusters are located on plasmids, whereas in *E. faecalis* CQNBZ21‐135, the *eps* cluster is inserted in the chromosome flanked by MGEs. This strongly suggests that these clusters were acquired through HGT. Conversely, in *E. hirae* FDAARGOS_1057, the *eps* cluster was located in the chromosome but showed no association with mobile elements or HGT markers, suggesting it is native to this strain. Thus, it may represent the origin of *eps* genotype VII. Notably, aside from the *eps* clusters themselves, these *Enterococcus* strains did not share sequence similarity in their adjacent regions with the *Pseudoactococcus* strains analyzed in this study (Figure 3).

### 3.5 Genetic Context of the *cwps* Gene Clusters

Unlike the *eps* clusters, the *cwps* clusters were not identified as part of genomic islands and appear to be stably located within the chromosomes, consistent with previous findings in *L. lactis* and *L. cremoris* (Mahony et al. 2020). These species generally harbor the *cwps* clusters in positions ranging from ~150,000 bp to ~250,000 bp within the chromosome, similar to most *P. raffinolactis* strains analyzed in this study and *P. laudensis* DSM 28961 and T2E11. However, some intrachromosomal mobility was observed, as described earlier, where transposase insertions caused genomic rearrangements in *P. laudensis* T2H4 and T2G5 (Table 1).

All *P. laudensis* and *P. raffinolactis* strains share common genes flanking their *cwps* clusters. In the region upstream of the predicted *cwps* locus, these are genes that encode proteins that may assist in CWPS biosynthesis and cell function but are not part of the primary biosynthetic functions, including a DUF4649 domain‐containing protein (annotated as ribose‐phosphate pyrophosphokinase in some Firmicutes), a 30S ribosomal protein, and a translation elongation factor, likely ensuring efficient protein synthesis. Additionally, a septation ring regulator (*ezrA*) suggests coordinated CWPS integration into the peptidoglycan layer during cell division. Downstream genes may aid CWPS maturation and cell wall integration, including molecular chaperones (*groES* and *groEL*), which ensure proper protein folding, and genes encoding endo‐β‐N‐acetylglucosaminidase, glutamyl aminopeptidase, and CHAP domain‐containing proteins, likely involved in cell wall remodeling.

Notably, the flanking regions of *P. raffinolactis* and *P. laudensis cwps* loci differ significantly from those in *L. lactis* IL1403, UC509.9, KLDS 4.0325, and *L. cremoris* NZ9000 (used as reference strains for the species). Apart from a ribosomal protein gene upstream and an amidase (N‐acetylmuramoyl‐l‐alanine amidase) downstream, no other genes are apparently sharing functionality. Common genes in *L. lactis* and *L. cremoris* strains include those predicted to encode FeoA and FeoB iron transporters, a metalloprotease, an IMP dehydrogenase, and a NadD nicotinate‐nucleotide adenylyltransferase, among others.

### 3.6 Structure of Cell Surface‐Associated Polysaccharides

#### 3.6.1 *P. laudensis* DSM 28961

To establish the chemical structure of cell surface‐associated polysaccharides (i.e., CWPS, CPS, and EPS) in emerging dairy pseudolactococcal species, the polysaccharides of the reference strains *P. laudensis* DSM 28961 and *P. raffinolactis* DSM 20443 were extracted.

Fractions obtained after separation of the different extracts of *P. laudensis* DSM 28961 on an anion‐exchange column contained l‐dTal, d‐Glc, and d‐Gal in different ratios. The absolute configuration of 6‐deoxytalose was determined as the l‐form by GC‐MS of the acetylated (R)‐(‐)‐2‐butyl derivative by comparison with the authentic standard, 6‐deoxy‐d‐talose from the O‐acetylated PS from *Pseudomonas (Burkholderia) plantarii* DSM 6535 (Zähringer et al. 1997) (Supporting Figure S3).

**Figure 4:**
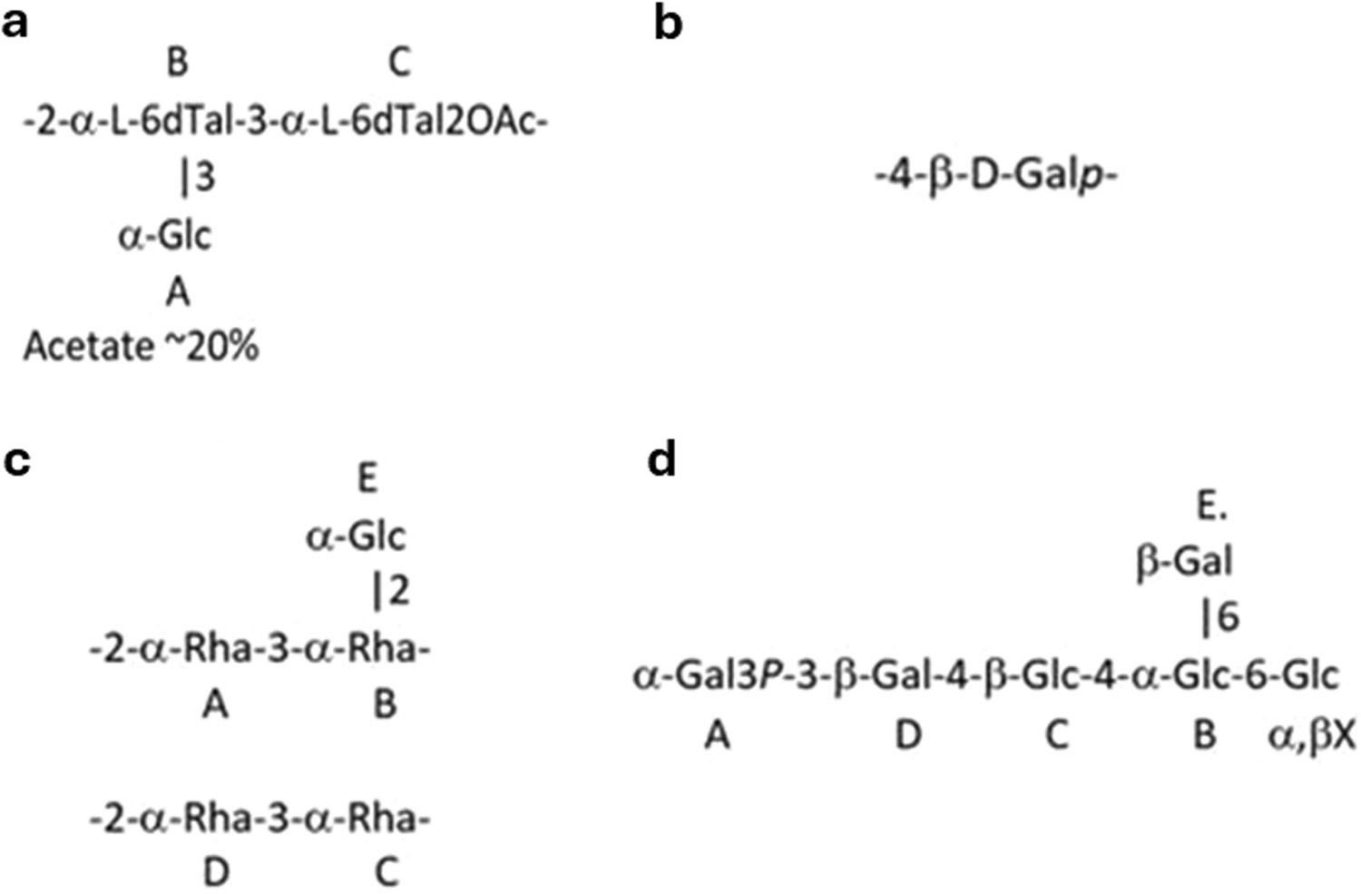
CWPS structures. (a) CWPS1 and (b) CWPS2 in *P. laudensis* DSM 28961; (c) Rhamnan and (d) PSP moieties in *P. raffinolactis* DSM 20443.

2D NMR analysis allowed to identify two distinct CWPSs: CWPS1, containing a relatively rare monosaccharide 6‐deoxy‐l‐talose, and a β‐(1‐4)‐galactan, CWPS2 (Figure 4ab, Supporting Table S2, Fig. S4). CWPS1 was found to be identical to the polysaccharide from *Bifidobacterium breve* 7017 (Manuscript in preparation), differing only by the degree of *O*‐acetylation (100% acetate in *B. breve* 7017% and 20% in *P. laudensis* DSM 28961, Supporting Figure S4 *)*. Upon deacylation, polysaccharides from both species produced an identical polysaccharide product.

CWPS2 is a common polysaccharide in plants, typically encountered as a side chain of rhamnogalacturonan I (Liwanag et al. 2013). Some bacteria such as *Bacillus licheniformis* can metabolize this polysaccharide (Ryttersgaard et al. 2004), yet to the best of our knowledge, it has not been previously reported as a bacterial CWPS.

#### 3.6.2 *P. raffinolactis* DSM 20443

*P. raffinolactis* 20443 was subjected to consecutive extractions, followed by fractionation on a Sephadex G50 column. HMW fractions from all extracts had similar ^1^H NMR spectra, although they did not look like a pure single (homogeneous) compound. These fractions contained rhamnose and glucose and were designated here as rhamnan.

NMR spectra of the rhamnan, that is, Correlated Spectra (COSY), Total Correlated Spectroscopy (TOCSY), Rotating frame Overhauser Effect Spectroscopy ROESY, ^1^H‐^13^C Heteronuclear Single Quantum Coherence (HSQC), and ^1^H‐^13^C Heteronuclear Multiple Bond Correlation (HMBC), contained several spin‐systems of α‐rhamnose and one α‐glucose (all in pyranose form). 2D ^1^H‐ and ^13^C spectra were fully assigned (Supporting Table S3, Fig. S5). The following Nuclear Overhauser Effect (NOE) cross‐peaks were observed: A1:B1,2,3,4,5; B1:A2; B1:E1; C1:D2; D1:C3 and HMBC: A1:B3; B1:A2; C1:D2; D1:C3 cross‐peaks were observed. This allowed us to conclude that there were two main repeating units, differing by the presence of glucose side chains (Figure 4c). There was approximately twice as much of the branched structure as the linear structure.

Methylation analysis of the rhamnan indicated the presence of 2‐, 3‐, and 2,3‐linked Rha and terminal Glc, in agreement with the structure established by NMR.

These units could be present in one or different PS chains, but there were no indications to decide what is correct. Spectra contained many small rhamnose signals, which could be due to linkages between different repeating units.

The structure of the rhamnan is essentially identical to one of the rhamnose‐glucose polysaccharides from *Bifidobacterium breve* 2003 and its EPS^‐^ mutant derivatives (Manuscript in preparation), except for the Glc residue E (Figure 4d), which is in an α‐configuration in *P. raffinolactis* and in a β‐configuration in *B. breve*.

The LMW product OS1, obtained from the AU extract, contained only Glc and Gal and had a distinct ^1^H‐NMR spectrum compared to the rhamnan. NMR analysis of the OS1 extract using 2D methods (COSY, TOCSY, ROESY, ^1^H‐^13^C HSQC, ^1^H‐^13^C HMBC, ^1^H‐^31^P HMQC) showed that it was a hexasaccharide with a mixture of α‐ and β‐Glc at the reducing end (Supporting Figure S6 and S7). To remove signal duplication due to anomeric configurations of Glc X, OS1 was reduced with NaBD_4_ to yield OS1red and analyzed by NMR (Fig. S6, Table S3). The following transglycoside NOE and HMBC correlations were observed: A1:D3; B1:A6, C1:B4, D1:C4; E1:B6 (Supporting Figure S7). This ultimately allowed the determination of the sequence of saccharides as presented in Figure S6.

OS1 contained a phosphate ester, which yielded a ^31^P signal at 1.72 ppm. It correlated with the A‐3 proton, indicating phosphorylation at terminal α‐Gal A3. The composition of the OS was confirmed by positive ion mode ESI MS (for the reduced with NaBD_4_ OS MS calc. 1073, observed neg. ion 1071.9 amu). We assume that OS1 represents the repeating unit of the PSP. Similar to the recently described PSP of *L. lactis* Tempeh6L (Parlindungan et al. 2024), the repeating units are most probably linked via an acid‐labile glycosyl phosphate‐phosphodiester bond between Glc F and α‐Gal A at position 3 (F‐1‐P‐3‐A). Upon cleavage of the unstable glycosyl phosphate bond, the phosphate group remains at position 3 of the α‐Gal A. Anomeric configuration of glycosylphosphates in *Lactococcus* polysaccharides is usually α, which is presented in the proposed structure of the original polysaccharide (Figure 4d and Supporting Figure S6).

### 3.7 Establishing the Genotype‐Structure Links

Previous studies (Mahony et al. 2020; Parlindungan et al. 2024; Theodorou et al. 2019) explored the relationship between lactococcal *cwps* loci and CWPS structures, paving the way for future structure prediction efforts. This study integrates genetic and chemical analyses of *P. laudensis* DSM 28961 and *P. raffinolactis* DSM 20443 to link *cwps* genes to their corresponding CWPS structures.

**Figure 5:**
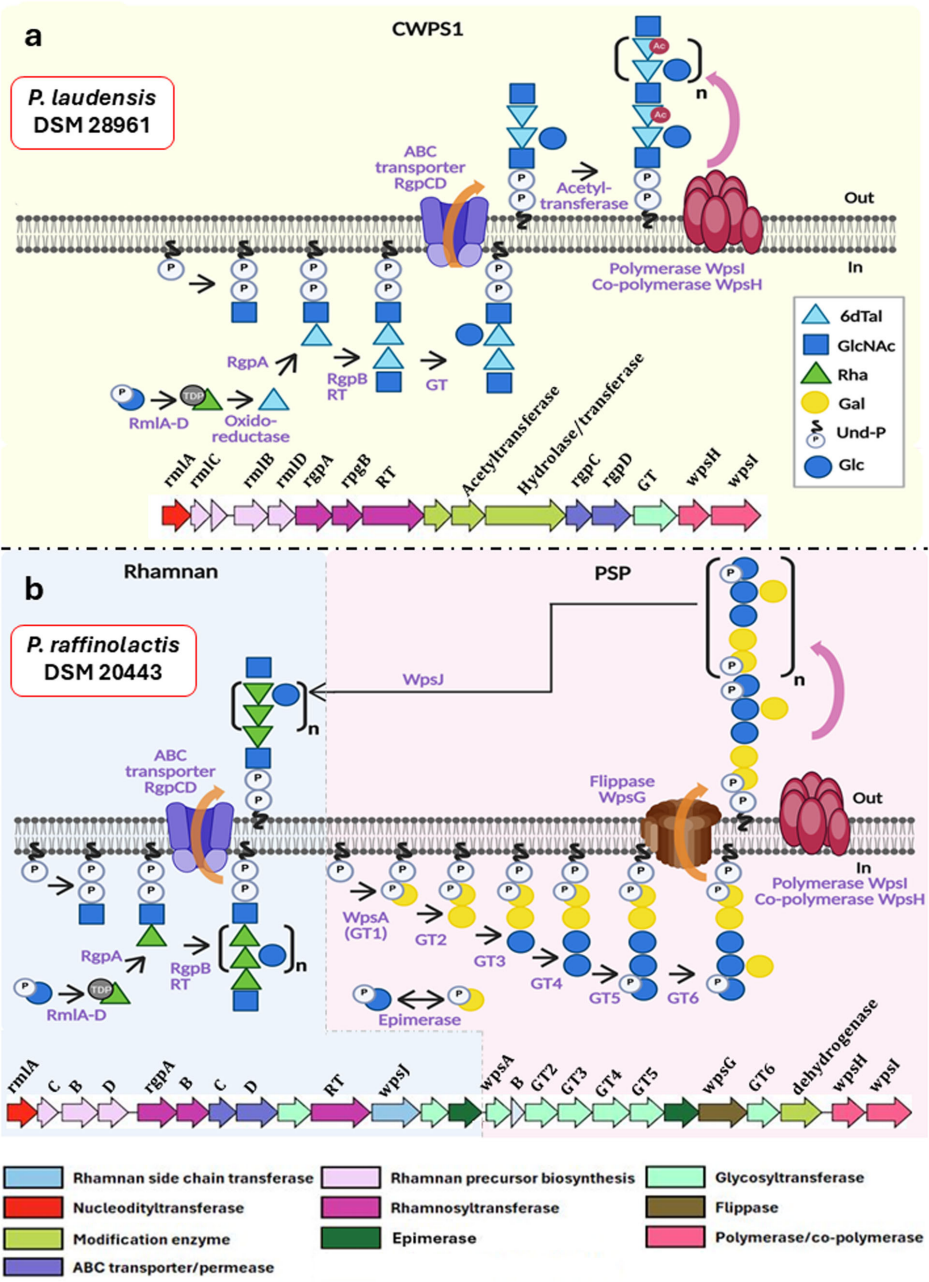
Models of heteropolysaccharidic CWPSs biosynthesis in (a) *P. laudensis* DSM 28961 and (b) *P. raffinolactis* DSM 20443. The proposed pathways remain to be validated by mutational analyses. Abbreviations: Ac, acetyl group; 6dTal, 6‐deoxy‐talose; Gal, galactose; Glc, glucose; GlcNAc, N‐acetylglucosamine; GT, glycosyltransferase; P, phosphate; Rha, rhamnose; RT, rhamnosyltransferase; TDP, thymidine diphosphate; Und‐P, undecaprenyl phosphate. Figure created with BioRender.

In *P. laudensis* DSM 28961, two independent CWPS structures, a 6‐deoxy‐αL‐talan (CWPS1) and a β‐(1,4)‐galactan homopolysaccharide (CWPS2), were identified. The CWPS1 structure resembles a lactococcal type B disaccharide rhamnan structure with a glucose side chain. The corresponding *cwps* locus contains the conserved lactococcal dTDP‐Rha precursor genes *rmlABCD, and* the rhamnosyltransferases *rgpAB*, followed by an additional rhamnosyltransferase and a NAD+‐dependent oxidoreductase, annotated as dTDP‐6‐deoxy‐l‐talose 4‐dehydrogenase (EC 1.1.1.134). This enzyme catalyzes a reversible reaction, interconverting dTDP‐6‐deoxy‐β‐l‐talose and dTDP‐4‐dehydro‐β‐l‐rhamnose (Mäki and Renkonen 2004), explaining the final 6‐deoxy‐α‐l‐talan structure (Figure 5a). Furthermore, a transmembrane protein with 10 TMHs was identified as an OafA superfamily acetylase by CDD, explaining the observed O‐acetylation at the C moiety (Figure 4a). The last modification enzyme, annotated as a sulfatase‐like hydrolase/transferase with 11 predicted TMHs, requires further investigation to elucidate its specific function. The glucose side chain could be either added by the remaining glycosyltransferase in the *cwps* locus (Figure 5a) or by other mechanisms, such as the TGS described for *L. cremoris* NZ9000 (Theodorou et al. 2020). In that sense, a homology search using nucleotide and protein BLAST was conducted to identify TGS candidates in the *P. laudensis* DSM 28961 genome, specifically homologs of the *L. cremoris* NZ9000 TGS genes, that is, *csdABCDEF* (Theodorou et al. 2020). The analysis revealed potential candidates, of which a system with a gene annotated as a putative membrane protein, followed by a hypothetical protein and a Bactoprenol glycosyltransferase, encoded in the chromosome in the positions 782310 to 785539 bp, is the most likely candidate. The Bactoprenol glycosyltransferase gene shares 56.3% amino acid sequence similarity and identical TMHs distribution with CsdA from *L. cremoris* NZ9000, whereas the putative membrane protein shares structure similarity with CsdB, but not sequence homology. Finally, the hypothetical protein possesses a structure resembling that of the WpsH co‐polymerase. Given that CsdAB is responsible for rhamnan glycosylation in *L. cremoris* NZ9000, it is reasonable to hypothesize that the described system in *P. laudensis* DSM 28961 may have the same role. Finally, the presence of genes resembling the YcaA‐like putative polymerase and WpsH‐like putative co‐polymerase from *L. lactis* and *L. cremoris* C and D genotypes is likely responsible for the talan polymerization (Figure 5a).

The β‐(1,4)‐galactan homopolysaccharide (CWPS2) is not linked to CWPS1, and that makes feasible that it is synthesized independently by genes encoded outside the *cwps* cluster. In *Lactobacillus johnsonii* FI9785, a bactoprenol glycosyltransferase is responsible for the synthesis of an EPS glucan homopolysaccharide (Mayer et al. 2020). Therefore, the system encoded in the positions 782310 to 785539 bp, described in the former paragraph, is also a likely candidate for the synthesis of this structure. Alternatively, CWPS2 could be synthesized by the glycosyltransferase within the *cwps* locus (Figure 4a), exported via the *rgpCD* ABC transporter, and polymerized by the YcaA‐like putative polymerase and WpsH‐like putative co‐polymerase. Experimental validation is necessary to confirm the genes encoding the synthesis of CWPS2.

The CWPS of *P. raffinolactis* DSM 20443 consists of a rhamnan and a PSP moiety, typical of lactococcal species, and aligns with the structure of its *cwps* locus. This locus is clearly divided into the PSP‐encoding region (starting from the priming glycosyltransferase WpsA) and the rhamnan‐encoding region (spanning from the RmlA nucleotidyltransferase up to, but excluding, WpsA) (Figure 5b).

The rhamnan of *P. raffinolactis* DSM 20443 is composed of a rhamnose disaccharide, resembling those of lactococcal B‐type strains (Vinogradov et al. 2018) and *Streptococcus thermophilus* UCC St50 and ST64987 (Lavelle et al. 2022; McDonnell et al. 2020). Furthermore, this rhamnan structure is eventually further decorated with a glucose side chain, likely added by a glycosyltransferase encoded within the rhamnan‐encoding region of the *cwps* cluster (Figure 5b) or, alternatively, by a TGS encoded elsewhere within the genome. Protein BLAST results revealed a glycosyltransferase in position 2041507 to 2042436 bp sharing 56.5% amino acid sequence similarity with the CsdA from *L. cremoris* NZ9000; however, no apparent candidates were found for a putative CsdB‐like encoding gene.

The PSP of *P. raffinolactis* DSM 20443 consists of a repeating hexasaccharide unit, composed of a linear pentasaccharide of galactose‐glucose with a galactose side chain. This hexasaccharidic structure matches the six glycosyltransferases, including the WpsA priming glycosyltransferase, present within the PSP‐encoding region of the cluster. This suggests that no TGS system located elsewhere within the genome has an influence on the final PSP structure, such as *csdCD* in *L. cremoris* NZ9000 (Theodorou et al. 2020). It is predicted that the glycosyltransferases encoded within this cluster sequentially add the individual saccharides to the growing PSP side chain subunits as has been proven in *L. lactis/cremoris* (Figure 5b); however, this requires validation through mutational analysis (Theodorou et al. 2019).

Regarding its glucose‐galactose composition, the NAD‐dependent epimerase within the cluster likely catalyzes the interconversion of UDP‐glucose to UDP‐galactose (Figure 5b), ensuring the availability of both monosaccharides required for PSP synthesis.

Finally, an unmatched feature is the phosphate group attached to the A and F moieties (Figure 4d). Given the lack of an apparent kinase or phosphotransferase within the *cwps* gene cluster, the most plausible explanation is that these galactose and glucose moieties are phosphorylated before their participation in the CWPS biosynthesis, where they are incorporated as such into the PSP.

## 4 Discussion

CWPSs are involved in several functions, including cell division and cell wall biogenesis, defense against phagocytosis, or bacteriophage adsorption (Chapot‐Chartier et al. 2010; Mahony et al. 2013, 2017; Theodorou et al. 2019), among others. Given these essential roles, it is not surprising that *cwps* clusters have been identified for the totality of studied *L. lactis* and *L. cremoris* up to date (Mahony et al. 2020; Parlindungan et al. 2024). The present study provides evidence for their presence in all analyzed *P. laudensis* and *P. raffinolactis* strains, suggesting that CWPSs have equally critical roles in these species. The *rmlABCD* and *rgpABCD* genes, located at the proximal end of the *cwps* clusters, that is, in the rhamnan encoding region, are highly conserved across *L. lactis* and *L. cremoris* strains. Our analysis revealed that these genes are conserved as well in *P. laudensis* and *P. raffinolactis*, confirming their utility as marker genes for identifying *cwps* clusters in other (pseudo)lactococcal species.

The current study identified eight novel *cwps* genotypes (E–L), significantly expanding the diversity of these gene clusters within the *Lactococcus* and *Pseudolactococcus* genera, which previously comprised four genotypes (A–D), including 11 subtypes within type C (C_1_‐C_11_) (Parlindungan et al. 2024).

The A–D genotypes feature a *cwps* cluster where the rhamnan and PSP components are biosynthesized and exported independently across the cell membrane, where they are subsequently assembled by WpsJ, as proposed in existing models (Mahony et al. 2020; Theodorou et al. 2019). Notably, a similar *cwps* organization was observed in all the newly identified *P. laudensis* and *P. raffinolactis* genotypes, except for E and G.

In the E and G *cwps* genotypes, the absence of genes encoding key PSP biosynthesis components, such as the priming glycosyltransferase WpsA, the assisting membrane protein WpsB, the WpsG flippase responsible for translocating the PSP to the external side of the cell membrane, and WpsJ, which likely attaches the PSP to the rhamnan component (Theodorou et al. 2020), suggests a CWPS assembly pathway distinct from previously described *Lactococcus* models. It is reasonable to speculate that, in these strains, the entire rhamnan‐PSP structure may be assembled on the inner side of the cell membrane and subsequently transported as a complete unit via ABC transporters. Additionally, these *cwps* clusters encode fewer glycosyltransferases, indicating a less complex side chain. Interestingly, in the type E strains, only a single glycosyltransferase was identified alongside the encoded rhamnosyltransferases, raising the possibility of an absent PSP component.

Chemical analysis of the reference strain *P. laudensis* DSM 28961 (type E) revealed the presence of two distinct CWPSs: a 6‐deoxy‐α‐l‐talan polysaccharide (CWPS1) and β‐(1,4)‐galactan homopolysaccharide (CWPS2). These findings represent novel discoveries in (pseudo)lactococcal CWPS structures. First, CWPS1 replaces the rhamnan component typically observed in *Lactococcus* strains with a 6‐deoxy‐α‐l‐talose‐containing backbone. This substitution highlights a unique genetic or enzymatic adaptation within type E strains. Second, CWPS2 is a homopolysaccharide, contrasting with all previously described lactococcal CWPS, which are characterized by heteropolysaccharidic structures. Finally, the structural independence of CWPS1 and CWPS2 confirms the absence of a typical PSP in *P. laudensis* DSM 28961. In the classical definition of CWPS, the polysaccharide pellicle (PSP) acts as a side chain attached to the rhamnan core. Here, the two polysaccharides are not covalently linked, confirming a distinct mechanism of CWPS biosynthesis and assembly in type E strains. Additionally, this study also provided the CWPS structure of the reference *P. raffinolactis* DSM 20443 (type I), confirming its classical rhamnan‐PSP structure and increasing the ever‐expanding catalog of (pseudo)lactococcal CWPS compositions (Supporting Tables S4 and S5).

Recent studies (Mahony et al. 2020; Parlindungan et al. 2024; Theodorou et al. 2019, 2020) have established the foundation for CWPS structure predictions based on the genetic composition of the *cwps* loci. The present study contributes to this growing knowledge base by identifying heretofore undiscovered genotypes and resolving novel CWPS structures. Lactococcal phages primarily utilize the PSP as a receptor for identifying and binding to their hosts (Mahony et al. 2017). Therefore, accurate prediction of CWPS, particularly of their PSP region, would represent a significant step toward palliating fermentation failures in the dairy industry caused by phage infections. Notably, some lactococcal phages are known to utilize receptors other than CWPSs, namely membrane proteinaceous receptors in ceduoviruses (Millen and Romero 2016) or other saccharidic structures such as CPSs, as have recently been described for certain P335 phages (Millen et al. 2022).

The findings of Millen et al. (2022) highlighted the importance of adopting a holistic approach when studying cell surface‐associated polysaccharides. In line with this, genomic inspection of *P. laudensis* and *P. raffinolactis* strains revealed *eps* loci for HePS production in 25 out of 28 strains, identifying 11 distinct genotypes (I–XI). These findings suggest that most of the analyzed strains are producers of either EPS or CPS. Notably, all *eps* clusters were shown to be located within the chromosomes of these strains.

Further genomic analysis revealed the HGT origin of the *eps* clusters, which apparently had been integrated into the chromosomes as part of genomic islands on recombination hotspots or putative grounded prophages. These regions are known to be hubs for the integration of defense islands, antimicrobial resistance genes, and other genes conferring advantageous traits for their bacterial hosts (Ramisetty and Sudhakari 2019), in this case, EPS or CPS biosynthesis. Additionally, this study highlights the mobility of *eps* clusters, providing evidence of inter‐genera HGT between enterococcal and type VI and VII *P. laudensis* strains. Moreover, it unraveled the presence of what appears to be an ABC transporter‐dependent pathway for the synthesis of EPS or CPS for type X and XI *P. raffinolactis* strains, a pathway typically exclusively associated with CPS production in Gram‐negative bacteria (Schmid 2018).

Under the growth and extraction conditions applied to *P. laudensis* DSM 28961 and *P. raffinolactis* DSM 20443, no significant yields of either EPS were observed; therefore, their structures could not be resolved. Since production of EPSs is growth‐condition dependent (Cui et al. 2023), further investigation is needed to ascertain the conditions supporting their production in these strains to elucidate later their chemical structures.

## 5 Conclusions

The current study has provided novel insights into the emergent dairy species *P. laudensis* and *PP. raffinolactis*, expanding the known genetic diversity and metabolic pathways for CWPS and EPS/CPS biosynthesis within the dairy‐associated representatives of the *Lactococcus‐Pseudolactococcus* clade. It also sheds light on the *cwps* and *eps* gene clusters and their genetic contexts, while presenting the first resolved CWPS structures for pseudolactococcal species, thereby laying down a solid foundation for future investigations.

These findings not only deepen our understanding of *Pseudolactococcus* cell surface‐associated polysaccharides but also offer valuable insights into microbial adaptation mechanisms and phage resistance, with significant potential for improving industrial fermentation systems.

Finally, further research should include targeted gene knockouts combined with structural analysis to validate the CWPS biosynthetic pathways. Furthermore, to strengthen the predictions of the activity of the specific glycosyltranferases (and other gene products), additional CWPS structures will need to be elucidated in the future. Additionally, transcriptomic studies performed under varied growth conditions will be essential to elucidate the regulatory mechanisms and the conditions driving EPS/CPS expression and production.

## Author Contributions

**Axel Soto‐Serrano:** writing – original draft, writing – review and editing, investigation, visualization, validation, formal analysis, software, project administration, data curation, methodology. **Irina Sadovskaya:** data curation, methodology, investigation, writing – review and editing, visualization, validation, formal analysis, resources. **Evgeny Vinogradov:** data curation, formal analysis, investigation, methodology, resources, writing – review and editing, validation. **Wenwen Li:** investigation, methodology, writing – review and editing. **Jun‐Hyeok Yu:** writing – review and editing, investigation. **Kelsey White:** investigation, writing – review and editing. **Douwe Sinderen:** writing – review and editing, resources, supervision, funding acquisition. **Lukasz Krych:** writing – review and editing, resources, supervision, methodology, software, funding acquisition. **Paulina Deptula:** funding acquisition, investigation, project administration, formal analysis, supervision, resources, data curation, validation, writing – review and editing. **Jennifer Mahony:** project administration, formal analysis, data curation, supervision, resources, methodology, validation, writing – review and editing, funding acquisition, investigation, conceptualisation.

## Ethics Statement

The authors have nothing to report.

## Conflicts of Interest

The authors declare no conflicts of interest.

## Supporting information


**Figure S1:** Generic dual chain assembly model for lactococcal CWPS assembly. **Figure S2:** Overview of the organization and sequence similarity of the *cwps* gene clusters of *P. laudensis* type F. **Figure S3:** Determination of the absolute configuration of 6dTal. **Figure S4:** Overlap of the HSQC spectra of the CWPS of *P. laudensis* DSM 28961 (green), *B. breve* 7017 (red), and O‐deacylated *B. breve* 7017 (black). **Figure S5:** ¹H–¹³C HSQC of the rhamnan from *P. raffinolactis* DSM 20443. **Figure S6:** Structure of the oligosaccharide products OS1 and OS1red from *P. raffinolactis* DSM 20443 and a proposed structure of PSP. **Figure S7:** HSQC spectrum of OS1 from *P. raffinolactis* DSM 20443 (blue‐green) and HMBC correlations from the anomeric protons in magenta. “a” and “b” denote α‐ and β‐anomers, respectively. **Table S1:** Accession numbers. **Table S2:** ¹H and ¹³C NMR data (δ, ppm, D₂O, 25°C, 600 MHz) for the galactan from *L. laudensis* DSM 28961. **Table S3:** ¹H and ¹³C NMR data (δ, ppm, D₂O, 25°C, 600 MHz) for the rhamnan from *P. raffinolactis* DSM 20443. **Table S4:** NMR data for the free (OS) and reduced (OSred) oligosaccharides from *P. raffinolactis* DSM 20443 (δ, ppm; Bruker AVANCE III 600 MHz, 25°C). **Table S5:** Chemical structures of the rhamnans of selected lactococcal strains. **Table S6:** Chemical structures and characteristics of PSP components of *cwps* C‐ and D‐type lactococcal strains.

MO SupplementaryData 060825.

## Data Availability

All data supporting the findings of this study are available within the article and in the accompanying Supporting Materials and Supporting Data files.
