# Supplementary material for: “Expanding the Lactococcal Cell Wall Polysaccharide Paradigm: Novel Structures and Metabolic Pathways in the Emerging Dairy Species Pseudolactococcus laudensis and Pseudolactococcus raffinolactis”
Source: Microbiologyopen. 2025 Nov 10;14(6):e70133. doi: 10.1002/mbo3.70133 (PMC12599335; doi:10.1002/mbo3.70133)
Supplement: Supplementary file 1 — Figure S1: Generic dual chain assembly model for lactococcal CWPS assembly. Figure S2: Overview of the organization and sequence similarity of the cwps gene clusters of P. laudensis type F. Figure S3: Determination of the absolute configuration of 6dTal. Figure S4: Overlap of the HSQC spectra of the CWPS of P. laudensis DSM 28961 (green), B. breve 7017 (red), and O‐deacylated B. breve 7017 (black). Figure S5: ¹H–¹³C HSQC of the rhamnan from P. raffinolactis DSM 20443. Figure S6: Structure of the oligosaccharide products OS1 and OS1red from P. raffinolactis DSM 20443 and a proposed structure of PSP. Figure S7: HSQC spectrum of OS1 from P. raffinolactis DSM 20443 (blue‐green) and HMBC correlations from the anomeric protons in magenta. “a” and “b” denote α‐ and β‐anomers, respectively. Table S1: Accession numbers. Table S2: ¹H and ¹³C NMR data (δ, ppm, D₂O, 25°C, 600 MHz) for the galactan from L. laudensis DSM 28961. Table S3: ¹H and ¹³C NMR data (δ, ppm, D₂O, 25°C, 600 MHz) for the rhamnan from P. raffinolactis DSM 20443. Table S4: NMR data for the free (OS) and reduced (OSred) oligosaccharides from P. raffinolactis DSM 20443 (δ, ppm; Bruker AVANCE III 600 MHz, 25°C). Table S5: Chemical structures of the rhamnans of selected lactococcal strains. Table S6: Chemical structures and characteristics of PSP components of cwps C‐ and D‐type lactococcal strains. [file MBO3-14-e70133-s002.docx]

**SUPPLEMENTARY MATERIAL**

**
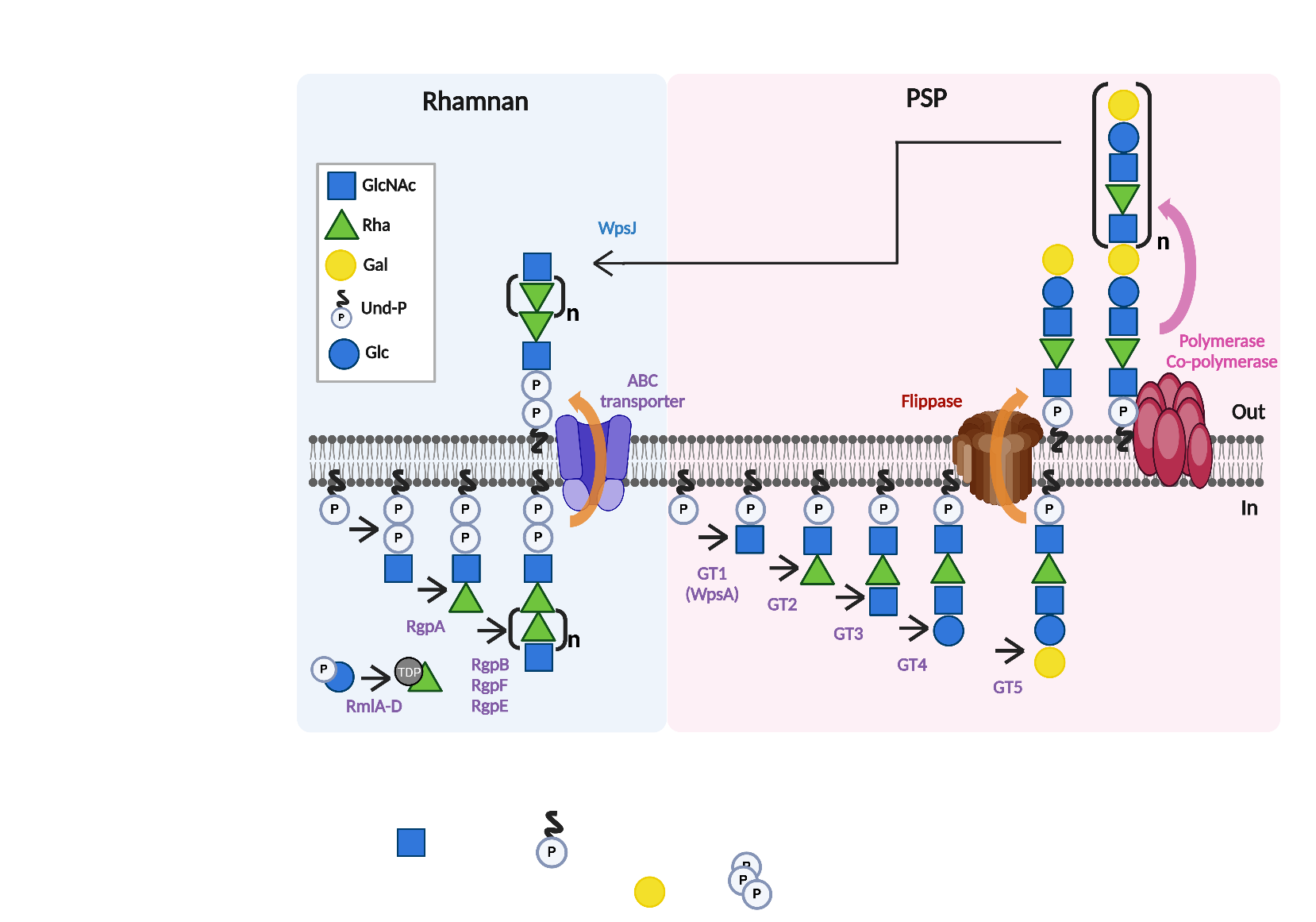
**

**Figure S1. Generic dual chain assembly model for lactococcal CWPS assembly**. Rhamnan synthesis occurs intracellularly, initiated by the addition of N-acetylglucosamine-1-phosphate (GlcNAc-P) to Undecaprenyl phosphate (Und-P) by TagO. The RmlABCD enzymes are responsible for synthesizing the rhamnose precursor, dTDP-L-Rha. Rhamnosyltransferase RgpA adds the first rhamnosyl residue to the GlcNAc-P-P-Und lipid intermediate, after which RgpB and RgpF extend the chain. Termination of the chain occurs with the addition of GlcNAc by RgpE. Finally, an ABC transporter system facilitates the transfer of the rhamnan chain to the outer side of the cytoplasmic membrane. PSP synthesis begins with the transfer of GlcNAc to Und-P by the priming GT WpsA (assisted by WpsB). The repeat unit is assembled on the GlcNAc-P-Und lipid precursor at the intracellular side of the cytoplasmic membrane through the sequential addition of monosaccharides by glycosyltransferases (GTs). The oligosaccharidic PSP repeat unit is then flipped to the extracellular side of the membrane by a flippase and polymerized by a polymerase and a putative co-polymerase and/or chain-length regulator.

**TableS1 – accession numbers**

| Species | Strain | Genome Accession nr | Biosample nr | Comments |
| --- | --- | --- | --- | --- |
| *P. laudensis* | DSM28961 | CP186774-CP186776 | SAMN46757937 |  |
| *P. laudensis* | T2A4 | CP186777-CP186780 | SAMN46757938 |  |
| *P. laudensis* | T2A6 | CP186781-CP186785 | SAMN46757939 |  |
| *P. laudensis* | T2A7 | CP186786-CP186788 | SAMN46757940 |  |
| *P. laudensis* | T2A8 | CP186789-CP186792 | SAMN46757941 |  |
| *P. laudensis* | T2C1 | CP186793-CP186796 | SAMN46757942 |  |
| *P. laudensis* | T2C6 | CP186797-CP186801 | SAMN46757943 |  |
| *P. laudensis* | T2C9 | CP186802-CP186805 | SAMN46757944 |  |
| *P. laudensis* | T2D8 | CP186806-CP186809 | SAMN46757945 |  |
| *P. laudensis* | T2E11 | CP186810-CP186814 | SAMN46757946 |  |
| *P. laudensis* | T2E12 | CP186815-CP186819 | SAMN46757947 |  |
| *P. laudensis* | T2E8 | CP186820-CP186822 | SAMN46757948 |  |
| *P. laudensis* | T2F10 | CP186823-CP186826 | SAMN46757949 |  |
| *P. laudensis* | T2F2 | CP186827-CP186832 | SAMN46757950 |  |
| *P. laudensis* | T2F8 | CP186833-CP186835 | SAMN46757951 |  |
| *P. laudensis* | T2G11 | CP186836-CP186838 | SAMN46757952 |  |
| *P. laudensis* | T2G3 | CP186839-CP186842 | SAMN46757953 |  |
| *P. laudensis* | T2G5 | CP186843-CP186846 | SAMN46757954 |  |
| *P. laudensis* | T2H1 | CP186847-CP186850 | SAMN46757955 |  |
| *P. laudensis* | T2H3 | CP186851-CP186855 | SAMN46757956 |  |
| *P. laudensis* | T2H4 | CP186856-CP186859 | SAMN46757957 |  |
| *P. raffinolactis* | WiKim0068 | CP023392.1 | SAMN07627433 |  |
| *P. raffinolactis* | DSM 20443 | CP048686.1 | SAMN14074143 | corresponding to KACC 14331 |
| *P. raffinolactis* | Lr_19_7 | CP047614.1 | SAMN13676123 |  |
| *P. raffinolactis* | Lr_19_5 | CP047616.1 | SAMN13676122 |  |
| *P. raffinolactis* | Lr_19_14 | CP047628.1 | SAMN13676120 |  |
| *P. raffinolactis* | Lr_18_12S | CP047630.1 | SAMN13676119 |  |
| *P. raffinolactis* | APC3967 | CP147857.1 | SAMN40419224 |  |


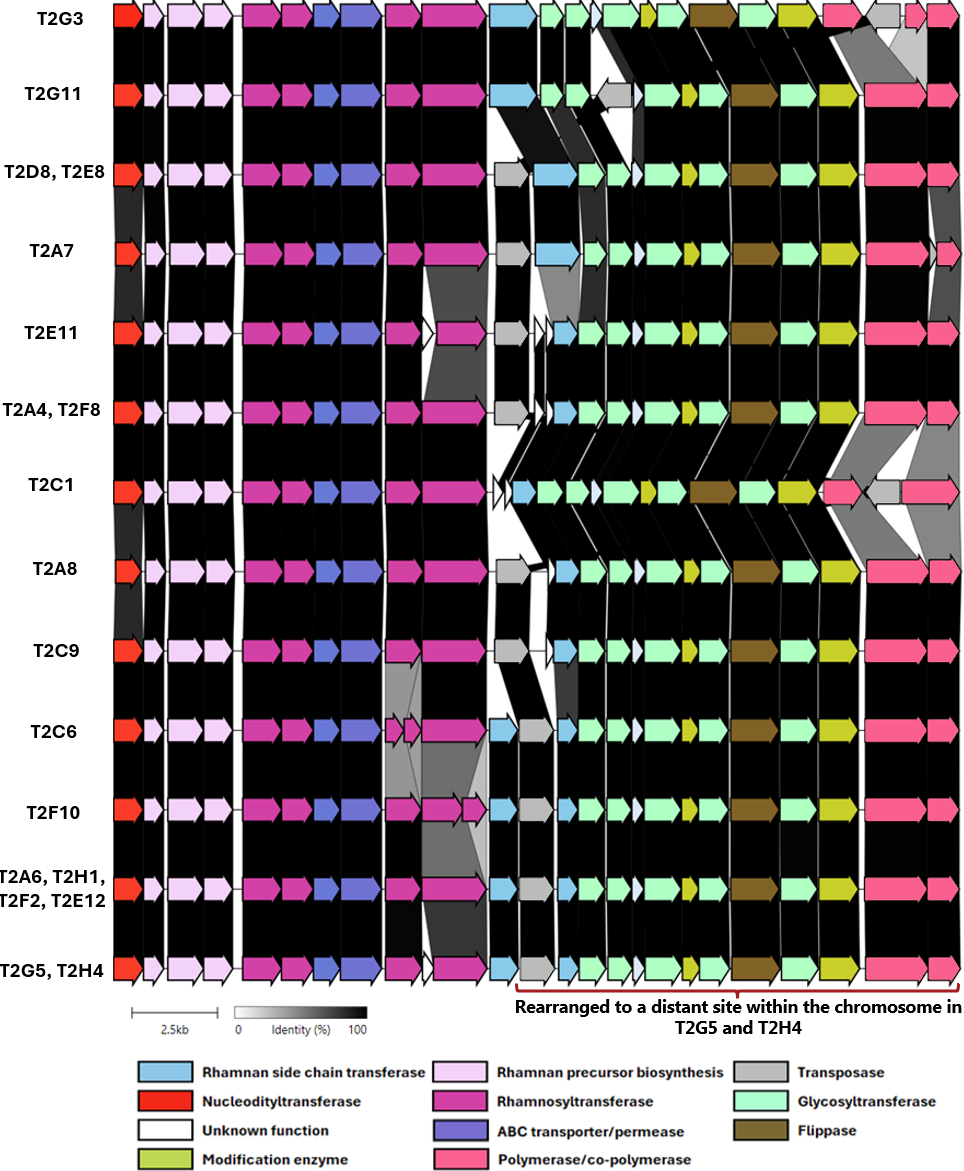


**Figure S2**. Overview of the organization and sequence similarity of the cwps gene clusters of *P. laudensis* type F.


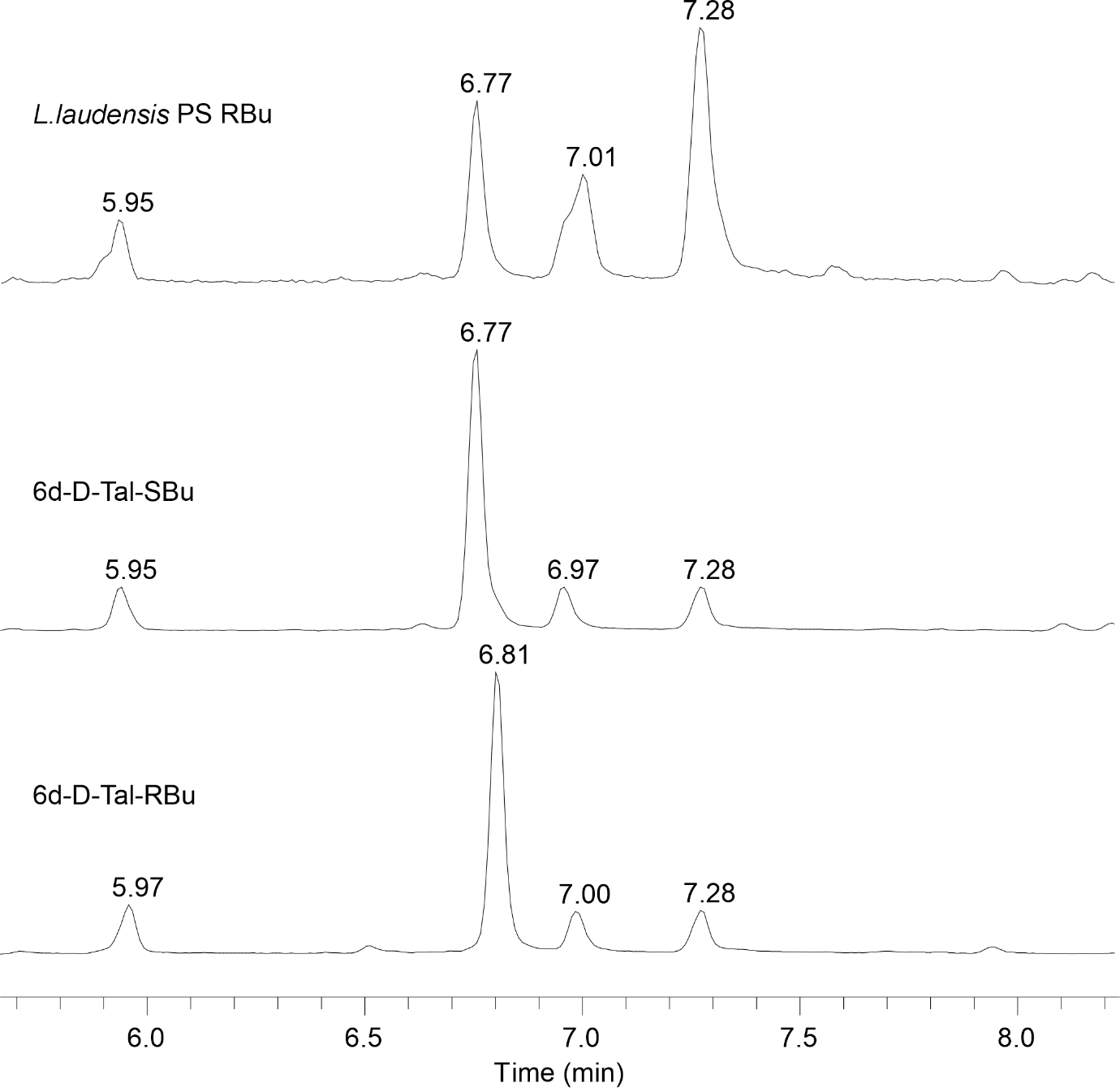


**Figure S3.** Determination of the absolute configuration of 6dTal.

**Table S2**. ^1^H and ^13^C NMR data (δ, ppm, D_2_O, 25°C, 600 MHz) for the galactan from *L.laudensis* DSM 28961.

| Sugar |  | H/C 1 | H/C 2 | H/C 3 | H/C 4 | H/C 5 | H/C 6 |
| --- | --- | --- | --- | --- | --- | --- | --- |
| -4-β-Gal- | H | 4.65 | 3.68 | 3.78 | 4.18 | 3.72 | 3.81; 3.81 |
|  | C | 105.5 | 73.0 | 74.4 | 78.8 | 75.6 | 61.9 |
| t-β-Gal- | H | 4.45 | 3.55 | 3.67 | 3.93 | 3.73 | 3.81; 3.81 |
|  | C | 104.0 | 72.1 | 73.7 | 69.8 | 76.6 | 61.9 |


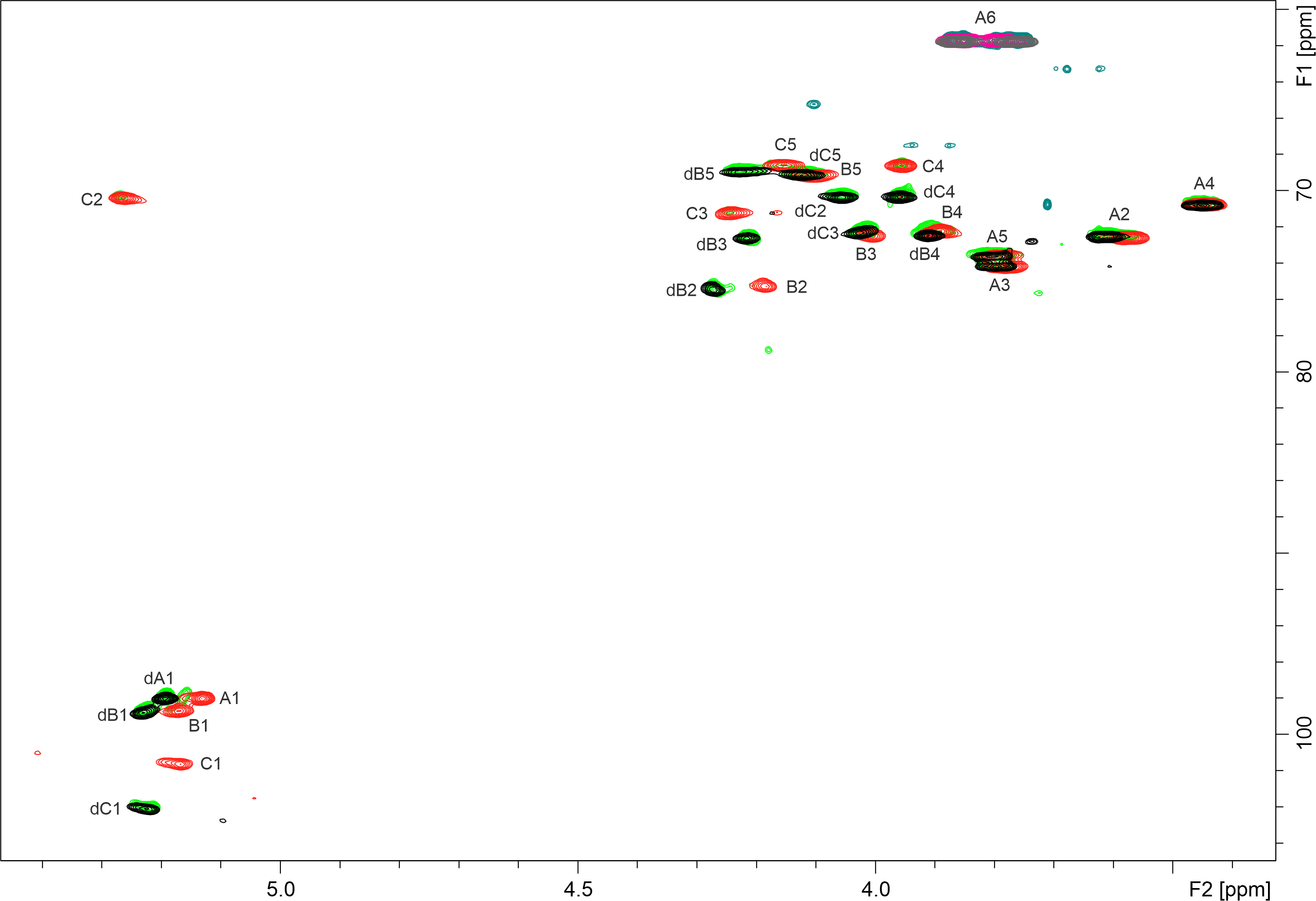


**Figure S4**. Overlap of the HSQC spectra of the CWPS of *P. laudensis* DSM 28961 (green), *B. breve* 7017 (red) and *O*-deacylated *B. breve* 7017 (black). Labels starting with "d" refer to deacylated compound, if deacylated and non-deacylated signals fully overlap "d" not used.

**Table S2**. ^1^H and ^13^C NMR data (δ, ppm, D_2_O, 25°C, 600 MHz) for the rhamnan from *P. raffinolactis* DSM 20443.

| Sugar |  | 1 | 2 | 3 | 4 | 5 | 6 |
| --- | --- | --- | --- | --- | --- | --- | --- |
| α-Rha A | H | 5.34 | 4.08 | 3.87 | 3.51 | 3.81 | 1.35 |
|  | C | 101.5 | 79.5 | 71.1 | 73.5 | 70.4 | 18.1 |
| α-Rha B | H | 5.11 | 4.20 | 4.07 | 3.73 | 3.78 | 1.29 |
|  | C | 100.5 | 77.1 | 75.6 | 73.6 | 71.1 | 17.6 |
| α-Rha C | H | 4.97 | 4.17 | 3.86 | 3.57 | 3.78 | 1.28 |
|  | C | 103.3 | 71.1 | 78.8 | 72.8 | 70.4 | 17.8 |
| α-Rha D | H | 5.20 | 4.09 | 3.96 | 3.51 | 3.84 | 1.33 |
|  | C | 102.1 | 79.5 | 71.0 | 73.5 | 70.4 | 17.9 |
| α-Glc E | H | 5.05 | 3.53 | 3.78 | 3.49 | 3.88 | 3.83; 3.86 |
|  | C | 98.8 | 72.6 | 73.7 | 70.7 | 73.4 | 61.7 |


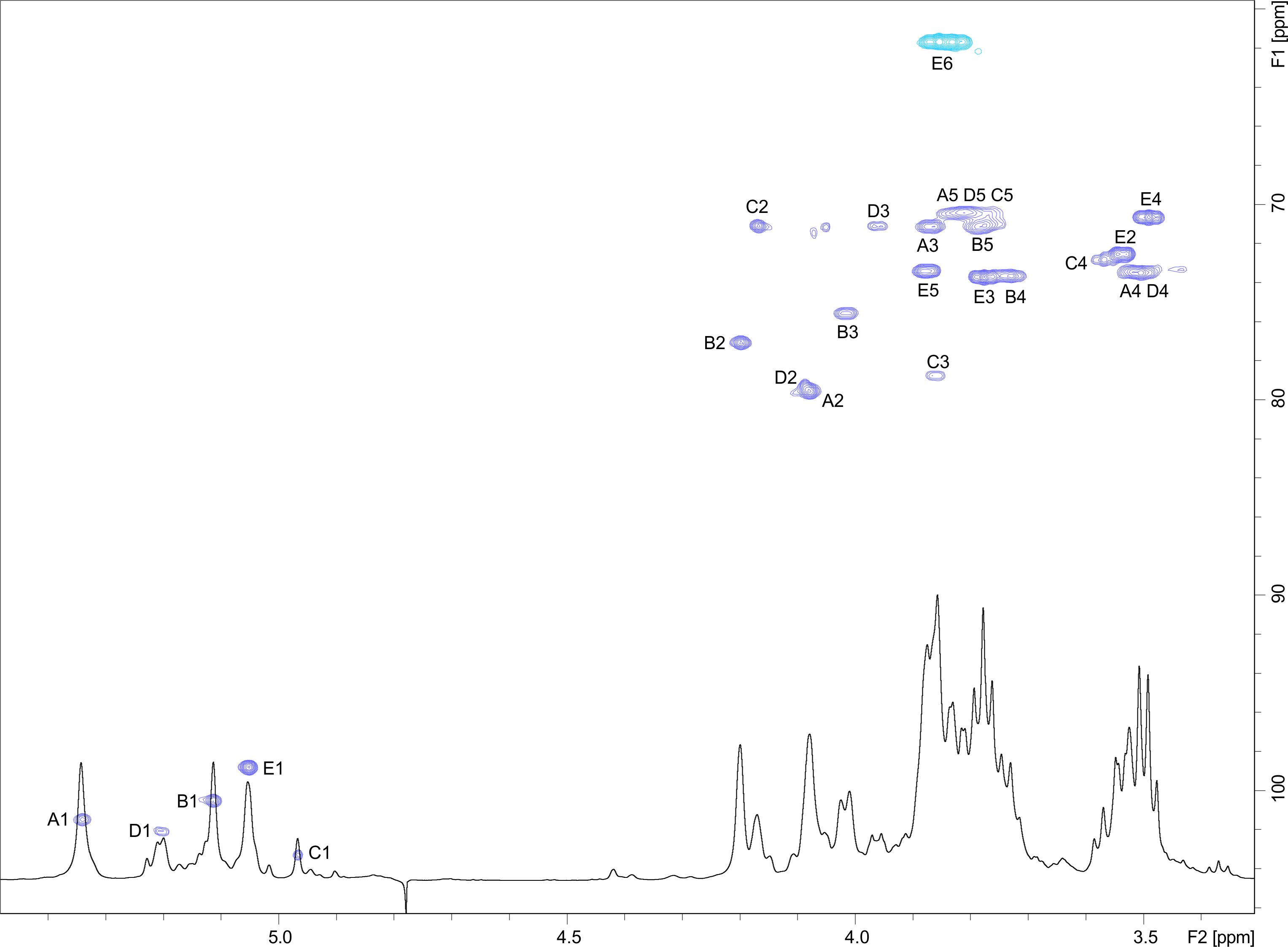


**Figure S5.** ^1^H-^13^C HSQC of the rhamnan from *P. raffinolactis* 20443

**Table S3**. NMR data for the free (OS) and reduced (OSred) oligosaccharides from *P. raffinolactis* DSM 20443 (δ, ppm; Bruker AVANCE III 600 MHz 25 °C).

| Sugar |  | H/C 1 | H/C 2 | H/C 3 | H/C 4 | H/C 5 | H/C 6 |
| --- | --- | --- | --- | --- | --- | --- | --- |
| α-Gal3P A | H | 5.19 | 4.01 | 4.42 | 4.24 | 4.24 | 3.76; 3.76 |
|  | C | 96.6 | 68.4 | 75.3 | 69.4 | 71.8 | 62.1 |
| α-Gal3P A' | H | 5.20 | 4.03 | 4.49 | 4.28 | 4.24 | 3.76; 3.76 |
|  | C | 96.6 | 68.4 | 76.1 | 69.4 | 71.8 | 62.1 |
| α-Glc B | H | 4.96 | 3.64 | 3.86 | 3.82 | 3.98 | 3.99; 4.25 |
|  | C | 99.2 | 72.4 | 72.7 | 79.0 | 70.5 | 68.1 |
| α-Glc B' | H | 4.98 | 3.61 | 3.86 | 3.82 | 3.98 | 3.99; 4.25 |
|  | C | 99.0 | 72.4 | 72.7 | 79.0 | 70.5 | 68.1 |
| β-Glc C | H | 4.63 | 3.38 | 3.69 | 3.69 | 3.63 | 3.82; 4.00 |
|  | C | 103.5 | 74.0 | 75.3 | 79.7 | 75.9 | 61.3 |
| β-Gal D | H | 4.53 | 3.71 | 3.79 | 4.19 | 3.71 | 3.77-3.82 |
|  | C | 104.1 | 70.6 | 78.5 | 65.9 | 76.3 | 62.2 |
| β-Gal E | H | 4.45 | 3.55 | 3.68 | 3.93 | 3.73 | 3.77-3.82 |
|  | C | 104.3 | 71.8 | 73.9 | 69.8 | 76.2 | 62.2 |
| α-Glc X | H | 5.24 | 3.54 | 3.70 | 3.51 | 4.01 | 3.73; 3.99 |
|  | C | 93.4 | 72.6 | 74.2 | 70.7 | 71.2 | 67.2 |
| β-Glc X | H | 4.67 | 3.26 | 3.48 | 3.51 | 3.64 | 3.79; 3.95 |
|  | C | 97.3 | 75.3 | 77.2 | 70.7 | 75.5 | 67.2 |
| Glc-ol X | H | 3.61; 3.73 | 3.84 | 3.87 | 3.73 | 3.94 | 3.69; 3.93 |
|  | C | 63.3 | 74.0 | 70.7 | 71.9 | 70.5 | 69.9 |
| α-Glc F | H | 5.59 |  |  |  |  |  |
|  | C | 96.3 |  |  |  |  |  |
| minor |  |  |  |  |  |  | 3.76; 4.04 |
|  |  |  |  |  |  |  | 66.6 |

E

β-Gal

|6

α-Gal3*P*-3-β-Gal-4-β-Glc-4-α-Glc-6-Glc OS1

A D C B α,β-F

E

β-Gal

|6

α-Gal3*P*-3-β-Gal-4-β-Glc-4-α-Glc-6-Glc-ol OS1red

A D C B F

E

β-Gal

|6

-3-α-Gal3*P*-3-β-Gal-4-β-Glc-4-α-Glc-6-α-Glc-*P*- PSP (proposed)

A D C B F

**Figure S6.** Structure of the oligosaccharide products OS1 and OS1red from *P. raffinolactis* DSM 20443 and a proposed structure of PSP.


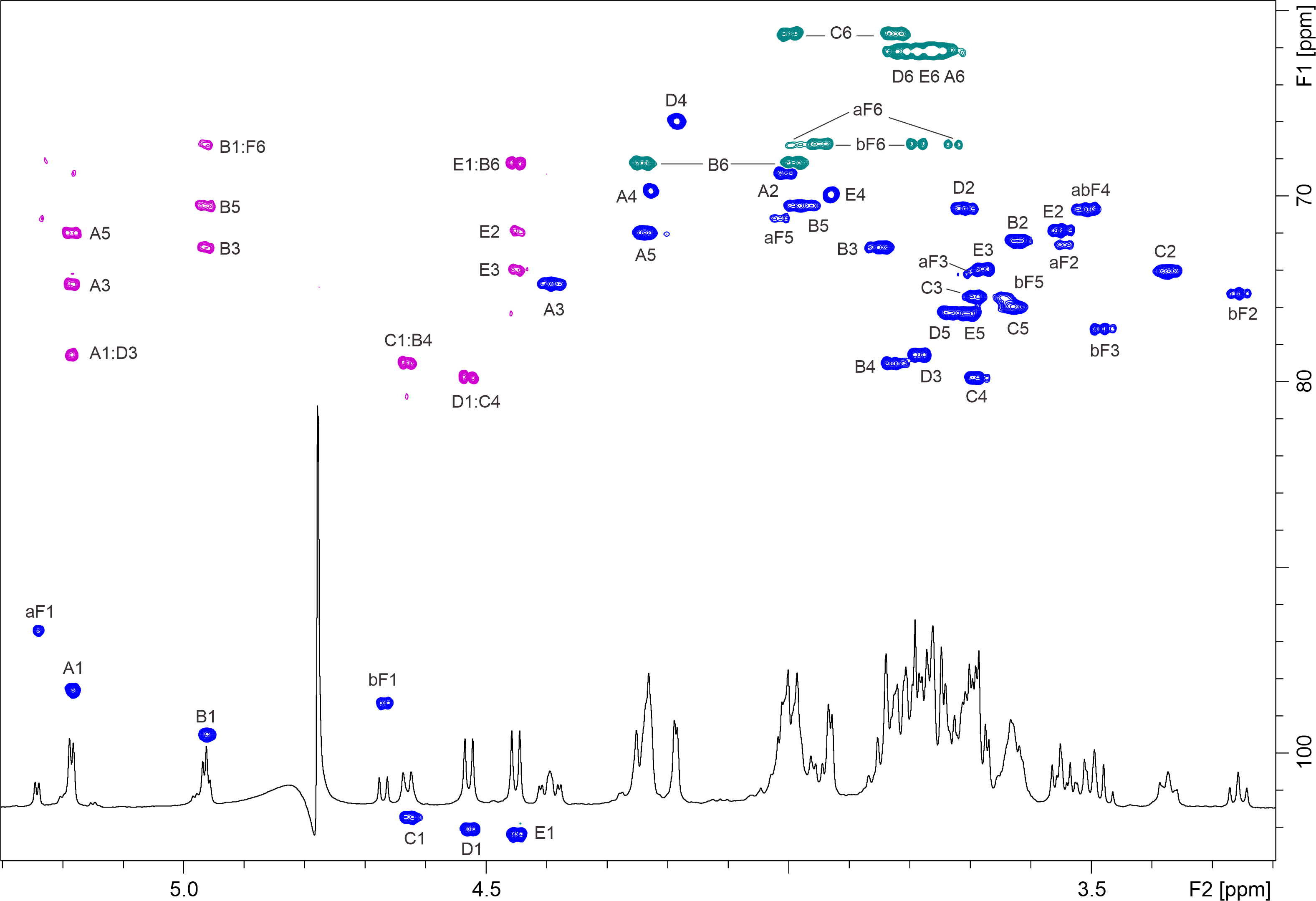


**Figure S7**. HSQC spectrum of OS1 from *P. raffinolactis* DSM 20443 (blue-green) and HMBC correlations from the anomeric protons in magenta. "a" and "b" are α- and β- anomers.

**Table S4.** Chemical structures of the rhamnans of selected Lactococcal strains

| **Strain** | **Rhamnan structure** | **Identical structure previously identified in strain** |
| --- | --- | --- |
| **RMN8G^#^** | -2-α-Rha-2-α-Rha-3-α-Rha- | MG1363, JM1*, 3107, W34, 1196 |
| **TolaII67^#^** | -2-α-Rha-2-α-Rha-3-α-Rha-  \|2  α-Glc | SK11 |
| **Tempeh6L^#^ (Rhamnan-1)** | -3-α-Rha-3-α-Rha-3-α-Rha-3-β-Glc-  \|2  α-Glc | N/A |
| **P. raffinolactis DSM 20443** | α-Glc  \|2  -2-α-Rha-3-α-Rha-  -2-α-Rha-3-α-Rha- |  |

**Table S5.** Chemical structures and characteristics of PSP components of *cwps* C- & D-type lactococcal strains.

| **Strain** | ***CWPS**  **type_subtype_** | **PSP subunit structure** | **Reference** |
| --- | --- | --- | --- |
| **MG1363** | **C_1a_** | -2-β-Gal*f*-6-α-Glc-*P-*6-β-GlcNAc-3-α-Rha-3-β-GlcNAc-  \|6  α-Glc | ([Chapot-Chartier et al., 2010](#_ENREF_7)) |
| **SMQ-388** | **C_1b_** | -2-β-Gal*f*-6-α-GlcNAc-*P*-6-α-GlcNAc-3-β-Gal*f*-3-β-GlcNAc-  \|6  α-Glc | ([Farenc et al., 2014](#_ENREF_11)) |
| **JM1** | **C_1c_** | -3-β-GlcNAc-  \|6  α-Glc | (Mahony et al. 2020) |
| **3107** | **C_2_** | -2-β-Gal*f*-6-α-GlcNAc-*P*-6-α-Glc-3-β-Gal*f-*3-β-GlcNAc- | ([Ainsworth et al., 2014](#_ENREF_1)) |
| **SK11** | **C_3_** | -6-β-Gal*f*-6-α-Gal-6-α-Glc-*P*-6-α-Gal-3-β-GlcNAc-  \|6  α-Glc | (Mahony et al. 2020) |
| **W34** | **C_4a_** | -6-β-Gal*f*-6-α-Glc*-*6-β-GlcNAc-3-β-Gal*f*-3-β-GlcNAc- | (Mahony et al. 2020) |
| **1196** | **C_4b_** | -6-β-Gal*f*-6-α-Gal*-*6-β-GlcNAc-3-β-Gal*f*-3-β-GlcNAc- | (Mahony et al. 2020) |
| **IO-1** | **C_5_** | -2-β-Gal*f*-5-Ara-ol-1-*P*-2-β-Gal*f*-3-α-GlcNAc-  \|3  α-GlcNAc | (Mahony et al. 2020) |
| **A76** | **C_6_** | -6-β-Gal-2-α-Gal-3-β-RhaOAc-4-β-Glc6*P*-6-β-Gal*f*-6-β-Glc- | (Mahony et al. 2020) |
| **184** | **D** | β-Glc  \|3  -5-Ribitol-1-*P*-6-α-GalNAc-3-β-GalNAc-  \|4  β-Glc*6OAla* | (Mahony et al. 2020) |
| **Tome1F^#^** | **C_7_** | β-Glc  \|3  -3-α-Rha-2-Glc-ol-6-*P*-6-β-Gal*f*-3-β-GlcNAc-  \|2 \|5  α-Gal β-GlcNAc | Parlindungan et al., 2024) |
| **RMN8G^#^** | **C_9_** | -2-β-Gal*f*-6-α-Glc-1-*P*-6-α-Glc-3-β-Gal*f*-3-β-GlcNAc-  \|2 \|6  α-Rha α-Glc | Parlindungan et al., 2024) |
| **Tempeh6L^#^** | **C_10_** | -3-α-Rha-2-α-Rha-*P*-6-β-Gal*f*-3-β-GlcNAc-  \|2 \|5  α-Gal-2-α-Rha α-Rha | Parlindungan et al., 2024) |
| **P. laudensis DSM 28961#** |  | -2-α-L-6dTal-3-α-L-6dTal2OAc(20%)-  \|3  α-Glc |  |
| **P. laudensis DSM 28961#** |  | -4-β-D-Gal- |  |
| **P. raffinolactis DSM 20443#** |  | β-Gal  \|6  -3-α-Gal3*P*-3-β-Gal-4-β-Glc-4-α-Glc-6-α-Glc-*P*- |  |

* Where CWPS elements of more than one strain of a given *cwps* genotype were assessed, the different resulting biochemical structures were assigned unique identifiers e.g. C_1a_, _b_, _c_ and C_4a_, _b_. ^#^ structure established in this study.
